# Liquid Sorption-Enhanced Haber–Bosch Process

**DOI:** 10.1021/acs.iecr.5c02713

**Published:** 2025-10-28

**Authors:** Nicholas E. Thornburg, Jacob H. Miller, William Xi, Hai Long, Kathleen D. Brown, Cheyenne Paeper, Rianna Martinez, Gabrielle A. Kliegle, Bryan S. Pivovar

**Affiliations:** 1 Energy Conversion and Storage Systems Center, 53405 National Renewable Energy Laboratory, 15013 Denver West Parkway, Golden, Colorado 80401, United States; 2 Catalytic Carbon Transformation and Scale-up Center, 53405 National Renewable Energy Laboratory, 15013 Denver West Parkway, Golden, Colorado 80401, United States; 3 Strategic Energy Analysis Center, 53405 National Renewable Energy Laboratory, 15013 Denver West Parkway, Golden, Colorado 80401, United States; 4 Computational Science Center, 53405 National Renewable Energy Laboratory, 15013 Denver West Parkway, Golden, Colorado 80401, United States; 5 Chemistry and Nanoscience Center, 53405 National Renewable Energy Laboratory, 15013 Denver West Parkway, Golden, Colorado 80401, United States

## Abstract

The use of a liquid sorbent in a traditional Haber–Bosch process enables significant improvements in energy efficiency and potential cost savings for arguably the most important chemical process on the planet. The approach presented in this report employs an incompressible liquid sorbent that absorbs and releases ammonia (NH_3_) under specific conditions. To achieve this, we investigate reactions of ammonia and pure phosphoric acid (H_3_PO_4_, PA), which rapidly neutralize to form an equilibrated solution of monoammonium phosphate (MAP) and diammonium phosphate (DAP) that functions as a reversible and regenerable sorbent. Through intimate contact of the gas-phase Haber–Bosch reaction mixture with this liquid absorbent, complete equilibrium uptake may be achieved in an appropriately sized separator, and facile separation occurs through the use of independent liquid and gas phases. Following depressurization and release of the ammonia product, only the incompressible fluid needs to be repressurized and returned to the reactor. This study documents proof-of-concept absorption and desorption experiments carried out in 75 mL batch reactors, predominantly charged with precise MAP and DAP mixtures that equilibrate at process-relevant temperatures and pressures. We then assemble the first thermodynamic relationships that underlie this advantaged separation strategy, validated by reactive force field (ReaxFF) interatomic potential simulations, and benchmarked with traditional separation routes via process modeling and technoeconomic analysis. The scale of energy consumption in the century-old Haber–Bosch process is massive, and the elegant liquid sorption approach reported here offers opportunities to enhance its energy efficiency for the next frontier of ammonia synthesis.

## Graphical abstract

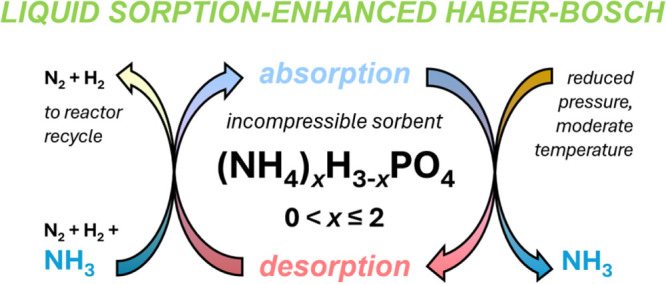

## 1 Introduction

While ammonia (NH_3_) is one of the most critical industrial chemicals for sustaining human life, the Haber–Bosch process for its production is also one of the highest-energy-consuming industrial processes in existence. Indeed, after 111 years of rigorous optimization, opportunities to improve energy efficiency^1^ and to reduce cost are scant due to the inability to implement further process intensification.^2^

The inherent technical challenges of Haber–Bosch lie in the need to achieve meaningfully fast catalytic rates via high reaction temperatures (400–550 °C), which introduces severe thermodynamic penalties—thus also requiring high pressures (150–300 bar) to shift equilibrium in favor of the NH_3_ product (eq 1).^3^

$$N_{2\left(g\right)}+3H_{2\left(g\right)}↔2{\mathrm{NH}}_{3\left(g\right)}$$ 1

The concomitantly low conversion-per-reactor-pass of hydrogen (H_2_) and nitrogen (N_2_) further necessitates enormous energy demands and staggering losses from NH_3_ condensation recovery^4–6^ and from the continuous decompression and recompression of reactant recycle loops^1^ that span 2 orders of magnitude in pressure change^3^—independently accounting for up to 22% of total energy inputs (5.5–6.5 GJ (LHV) t^–1^ NH_3_ of turbine and compressor demands alone!) and nearly 60% of all energy losses in modern Haber–Bosch plants.^1^ Therefore, novel, less energy-intensive ammonia separation techniques would enable immediate massive energy savings on commercial scales.

The vast majority of academic ammonia research emphasizes alternate synthesis routes,^7–13^ with relatively few studies that investigate ammonia separation technologies to address this important process footprint. Significant attention has been given to solid sorbents, including metal halides,^14–21^ metal hydrides,^21^ borohydrides,^14,21^ zeolites,^22,23^ metal/covalent organic frameworks,^24^ and membranes;^25^ each of these materials is proposed to integrate into ammonia synthesis loops via pressure and/or thermal swing ab-/adsorption beds, cartridges, or similar units operated cyclically to uptake and release the ammonia product.

However, known difficulties in handling solids in continuous chemical processes compel the development of liquid sorbents. Inspired by historical acid scrubbing^26^ approaches for trace NH_3_ gas removal, liquid sorption is an additional focus of the research literature, featuring several studies on ionic liquids^4,27–31^ and a few on concentrated acids.^32–34^ From our extensive search, only two studies quantify thermodynamics, one of ammonia–ionic liquid pairs^31^ and another of ammonium sulfates,^35^ essential information for equilibrium-staged unit operation design. However, significant toxicity^36^ and/or corrosivity^37^ concerns of these sorbents have likely precluded their industrial adoption.

The use of phosphoric acid (H_3_PO_4_, PA) and phosphate mixtures has been investigated for limited applications involving ammonia. The 1970s-era Phosam and Phosam-W processes^38–40^ enabled absorption and stripping of NH_3_ from coke-oven gases using an aqueous monoammonium phosphate ((NH_4_)­H_2_PO_4_, MAP) solution, forming a diammonium phosphate ((NH_4_)_2_HPO_4_, DAP) product and with subsequent thermal decomposition to release ammonia gas.^26,34^ A recent process modeling study simulated the application of the Phosam process for ammonia separation from small-scale modular Haber–Bosch plants, although additional water–ammonia separators are required following the thermal decomposition of DAP-rich solutions.^34^ There, water is also expected to generate significant vapor pressure in the desorption temperature regimes^41^ while contributing significantly to the heating requirements within sorbent heat exchange networks.^34,42^ In general, sorption-enhanced processes have been proposed for ammonia synthesis^41^ and for other processes^42^ but still require thermodynamic insights to mature. In all cases, key information about, and validation of, ammonia–phosphate equilibria is needed to guide unit operation and integrated process design and to enable reduction to practice of these thermodynamically limited separation approaches.

Additional inspiration may be drawn from first-generation phosphoric acid fuel cells, in which ammonia contaminants are well-known to strongly and selectively absorb.^43,44^ Advantageously, safe handling practices for low-water and anhydrous PA at elevated temperatures and pressures have been established by the fuel cell^45^ and PA manufacturing^37^ communities. When operating at low or near-zero water content, PA, MAP, and DAP feature essentially zero vapor pressure^34^ and are incompressible liquids above their melting points—making them excellent working fluids in a separation process. Beneficially, MAP and DAP are common fertilizer products, enabling the valorization of slipstream coproducts outside of closed-loop recirculation.

In this report, we seek to understand the liquid absorption and desorption of NH_3_ using synthetic phosphate sorbent mixtures as a potential reactive separation strategy for the Haber–Bosch process (Scheme 1). Our approach utilizes tandem acid–base chemistries at minimal water content for spontaneous NH_3_ absorption by PA (eq 2, forward reaction) and MAP (eq 3, forward reaction), generating a terminal DAP product that readily decomposes at elevated temperatures^34,35,46^ and/or reduced pressures to regenerate ammonia (eqs 2–3, reverse reactions).

$${\mathrm{NH}}_{3\left(g\right)}+H_{3}{\mathrm{PO}}_{4\left(l\right)}↔\left({\mathrm{NH}}_{4}\right)H_{2}{\mathrm{PO}}_{4\left(l\right)}$$ 2

$${\mathrm{NH}}_{3\left(g\right)}+\left({\mathrm{NH}}_{4}\right)H_{2}{\mathrm{PO}}_{4\left(l\right)}↔{\left({\mathrm{NH}}_{4}\right)}_{2}{\mathrm{HPO}}_{4\left(l\right)}$$ 3

**Scheme 1:**
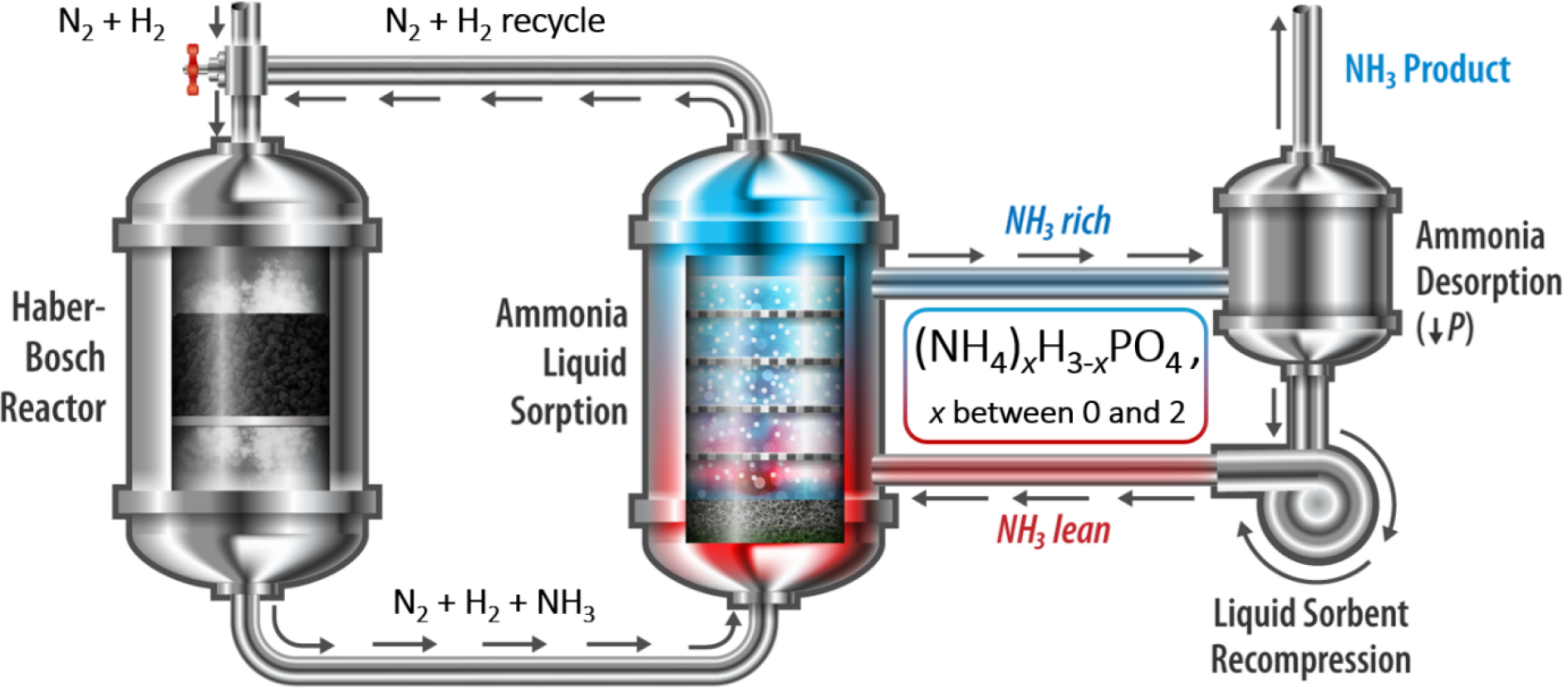
Simplified Liquid Sorption-Enhanced Ammonia Production Process^a^

a Proposed process concept involves a Haber–Bosch ammonia synthesis reactor (left) with an integrated, isobaric gas–liquid absorber column (center), an ammonia recovery unit (top right) and a recycle pump for the incompressible liquid sorbent (bottom right). The target sorbent is a mixture of PA, MAP, and DAP, represented by (NH_4_)*_*x*_* H_3–*x*_PO_4_, with 0 < *x* ≤ 2 yielding a combination of pure components PA (*x* = 0), MAP (*x* = 1), and DAP (*x* = 2) that varies from post-absorption (blue stream) to post-desorption (red stream). Process scheme is illustrative and not definitive. Multiple absorber designs, auxiliary unit operations, alternate contacting patterns and additional integration paths may be possible.

Analyzing headspace pressure evolution, we calculate batch ammonia release trends across equilibrated MAP and DAP mixtures charged at different temperatures and pressures, assembling the first thermodynamic relationships for this reactive separation system. The resulting single-stage equilibrium data sets and behaviors are further validated by simulations in custom-built reactive force fields (ReaxFF) for a model NH_3_–H_3_PO_4_ binary system, which demonstrate good agreement with desorption data under certain experimentally relevant regimes. Finally, an ammonia production process model is presented to estimate potential technoeconomic impacts of liquid sorption-based NH_3_ separation compared to traditional condensation approaches.

## 2 Experimental and Modeling Methods

### 2.1 Batch Sorption Experiments

Phosphoric acid solution (H_3_PO_4_, “PA”, 85 wt %, ACS reagent grade), anhydrous PA (crystalline, >99.999% trace metals basis), monoammonium phosphate ((NH_4_)­H_2_PO_4_, “MAP”, >99.99% trace metals basis; mp 190 °C^47^), diammonium phosphate ((NH_4_)_2_HPO_4_, “DAP”, >99.99% trace metals basis; mp (dec.) 155 °C^47^) and pyrophosphoric acid (H_4_P_2_O_7_, “PP”, technical grade) were obtained from Millipore Sigma. Deuterated water (D_2_O, 99.9% atom D) was obtained from Cambridge Isotope Laboratories, Inc. Trace-metal grade nitric acid (HNO_3_, <1 ppb metals assay) was purchased from Fisher Chemical. Research-grade anhydrous ammonia, ultrahigh-purity argon (Ar) and ultrahigh-purity helium (He) were purchased from MATHESON Tri-Gas, Inc. All reagents and gases were used as received.

Batch absorption and desorption experiments were carried out using a six-well Parr Instrument Company Series 5000 Multiple Reactor System (MRS; 300 °C MAWT, 3000 psig MAWP) equipped with six 75 mL batch reactors, magnetic stirring, thermowell temperature monitoring, a digital pressure transducer and a custom gas manifold. Each 75 mL vessel and all headpiece componentry were constructed of Hastelloy B2/B3 and sealed by PTFE gaskets. Borosilicate glass liners were inserted along the vessel inner walls prior to all fluid exposures. Leak tests were performed prior to all pressurized experiments with He using a Snoop surfactant-based leak detector (Swagelok Company). No quantifiable corrosion or degradation was observed in any experiment, although a slight discoloration of vessel internals was seen (Figure S1) after one extended use experiment.

Batch absorption experiments were conducted by charging 13.8 g anhydrous PA and 0.5 g PP to a 75 mL Parr vessel, followed by sealing, purging with Ar, venting to ambient pressure, and exposing the headspace to one or more doses of 3.8 bar NH_3_ without any external heating provisions. (We note that the local ambient pressure in Golden, CO, USA is 0.81 bar.) For sequential dosing experiments, pressure traces were allowed to equilibrate prior to charging the next dose of gas; pressure responses were monitored over several hours or days in some cases. Repeated NH_3_ dosing occurred until an equilibrium uptake was observed (i.e., negligible decrease in headspace pressure upon the final round of dosing).

Batch desorption experiments were executed by charging known masses of MAP and/or DAP to a 75 mL vessel (100:0, 75:25, 50:50, 25:75, and 0:100 MAP/DAP molar ratios with a total solids mass of approximately 15.0 g). After sealing, contents were magnetically stirred at 200 rpm, and the vessel headspace was purged and charged with 3–5 bar Ar, followed by heating to a specified temperature (25–250 °C). The final pressure was recorded after at least 6 h of equilibration at the target temperature before increasing the set point in 25 °C increments; this procedure was repeated until reaching a final temperature of 250 °C. For some experiments, 15.0 g DAP charges were thermally cycled between 200 and 250 °C for combined absorption and desorption trials; here, the reactor was initially purged and charged with 4.9 bar Ar before heating. We note that MAP and DAP remain partially solidified until 150 °C is attained (*vide infra*; onset of melt phases). MATLAB R2022a Curve Fitting Toolbox was utilized for parameter estimation.

### 2.2 Characterization

Thermogravimetric analysis (TGA) was performed using a TA Instruments TGA Q500 instrument with alumina pans in high-resolution mode using a temperature ramp from 20 to 750 °C at a rate of 1 °C min^–1^. Post-sorption liquids and solids were recovered for attempted compositional characterization by solution-phase ^31^P nuclear magnetic resonance (^31^P NMR), inductively coupled plasma optical emission spectroscopy (ICP-OES), elemental analysis (CHN) and powder X-ray diffraction (pXRD). ^31^P (128 scans) and ^1^H (16 scans) NMR spectra were collected on a 400-MHz Bruker Avance III spectrometer. ICP-OES triplicate analytes were dissolved in nitric acid, and spectra were measured on an Agilent 5110 ICP-OES. CHN content was measured on a LECO CHN628 instrument in triplicate analysis. XRD was performed on a Rigaku Ultima IV X-ray diffractometer with a Cu Kα radiation source. Diffraction patterns were collected in the 2θ range of 10–80°, with a step width of 0.04 and a scan speed of 5° min^–1^. Experimental diffraction peaks were identified using PDF-2 2021 software and database.

### 2.3 Molecular Modeling

Full details of molecular modeling procedures are reported in the Supporting Information (SI).^48–54^ Briefly, simulation boxes of different N:P ratios are generated using either *ab initio* molecular dynamics (AIMD) or reactive force field molecular dynamics (ReaxFF MD) simulations with a pre-equilibrated liquid phase and with a free space to allow NH_3_ to evaporate at different temperatures (Figure S2). Values for ammonia vapor pressure and Henry’s law constants were computed based on the relative concentrations of NH_3_ in the gas and liquid phases by counting the number of NH_3_ molecules within a specified volume from the MD simulation trajectories.

### 2.4 Process Modeling and Technoeconomic Analysis

Process models were developed in Aspen Plus software (version 14.0) using the Redlich–Kwong–Soave–Boston–Mathias (RKS–BM) thermodynamic property method, designed to handle mixtures of polar and non-polar vapors at elevated temperatures and pressures. Economic estimates utilized the centralized production model H2A^55^ modified for ammonia production within a discounted cash flow model. Full details of process modeling and technoeconomic analysis methods, assumptions, calculation bases and associated literature^20,56–61^ are described in the SI.

## 3 Results

### 3.1 Decomposition Behaviors

First, we sought to understand the thermal decomposition tendencies of target sorbent products MAP and DAP. We performed TGA under an air atmosphere on each pure compound to assess the desorption (i.e., release) of NH_3_ from these phosphate salts in otherwise ammonia-free environments. Mass loss and differential curves are displayed in Figure 1. Both compounds feature prominent peaks above 500 °C (MAP: 592 °C, DAP: 565 °C) in Figure 1b corresponding to decomposition of phosphoric acid, alongside broad peaks at ∼215 °C (MAP: 216 °C, DAP: 212 °C) corresponding to the release of one molecule of NH_3_. Additionally, a second peak at 165 °C for DAP corresponds to the loss of a second molecule of NH_3_. Taken together, these data affirm that two sequential reactions govern interconversions of PA, MAP, and DAP (eqs 2–3, reverse reactions). The location and size of these peaks match the theory reasonably well. The tabulated decomposition temperature^47^ of DAP is 155 °C, only 10 °C below the onset of our measurement. This lower-temperature decomposition peak of DAP comprises 12.5% of total mass loss, also agreeing well with a single NH_3_ molecule comprising 12.9% of the total mass of DAP. The ∼215 °C peaks for MAP and DAP both indicate the release of more mass than theoretical (MAP: 27.3% vs 14.8%; DAP: 24.9% vs 12.9%), although this is likely due to some extent of H_3_PO_4_ dehydrative dimerization to pyrophosphoric acid (H_4_P_2_O_7_, PP), followed by the dimer’s successive decomposition to phosphorus pentoxide (P_2_O_5_). These reactions are expected to occur over the broad temperature range of these peaks (188–439 °C) via sequential dehydration (eqs 4–5):

$$2H_{3}{\mathrm{PO}}_{4\left(l\right)}↔H_{2}O_{\left(g\right)}+H_{4}P_{2}O_{7\left(l\right)}$$ 4

$$H_{4}P_{2}O_{7\left(l\right)}↔2H_{2}O_{\left(g\right)}+P_{2}O_{5\left(s\right)}$$ 5

**Figure 1:**
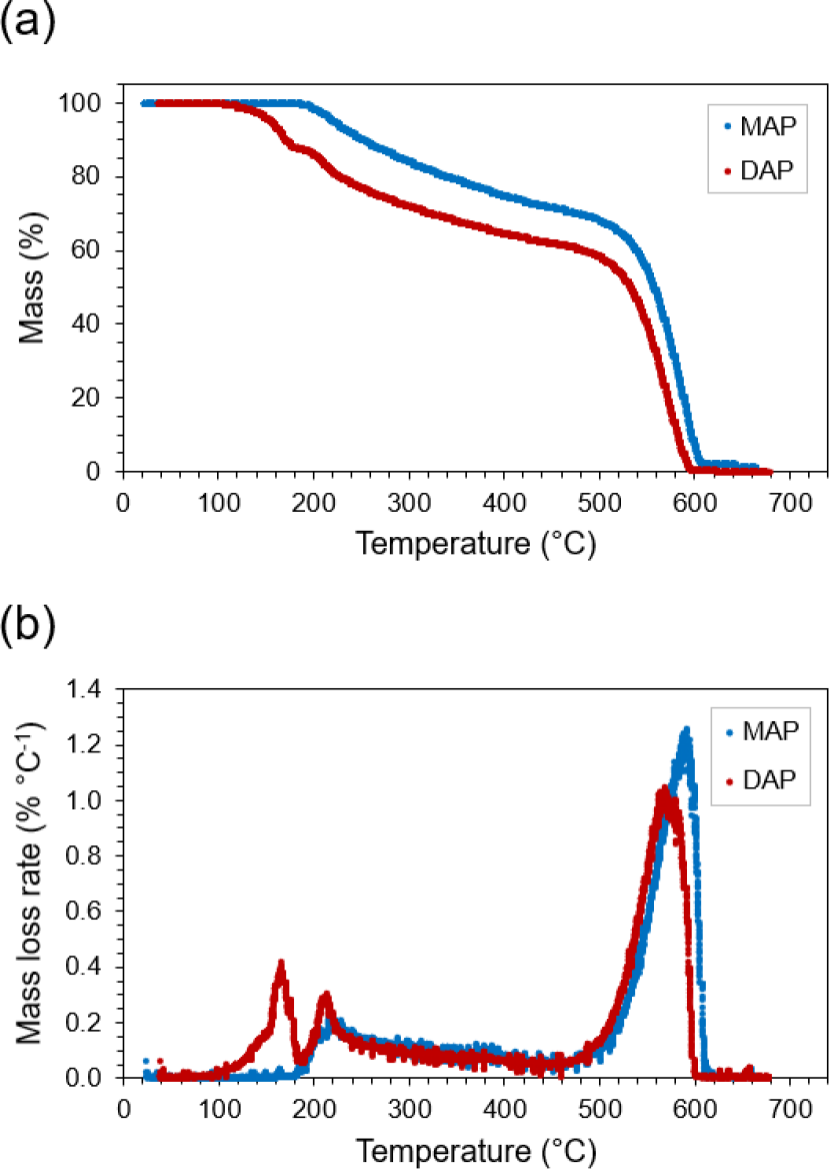
Thermogravimetric analysis (a) mass loss and (b) differential mass loss curves for ammonium phosphates MAP and DAP.

### 3.2 Ammonia Sorption by H_3_PO_4_

Next, we investigate sorption (i.e., uptake) behavior when NH_3_ gas is exposed to anhydrous PA. In early experiments with 85 wt % H_3_PO_4_ solutions, we found it difficult to decouple ammonia solubilization in water versus ammonia uptake via acid–base neutralization with PA. Further, at elevated temperatures, the volatilization of water overwhelmingly contributed to the measured headspace pressures across experimental temperature ranges. Taken together, we sought to obviate these complications by rigorously controlling for water content. Crystalline PA is commercially available, albeit hygroscopic; however, leveraging the interconversion of PA with its dimer PP, we titrate residual water in the crystalline PA reactant using a stoichiometric amount of PP^62^ (eq 4, reverse reaction, all liquid phases). ^1^H NMR confirms that the resulting water content is at most 0.1% (1000 ppm), on the order of the background proton signatures of D_2_O solvent; we note that Karl Fischer titration may not be suitable for this analyte. Thus, an appropriate mixture of PA and PP may then be exposed to NH_3_ vapor during sorption trials without the confounding effects of water.

Figure 2 illustrates the evolution of ammonia pressure over time during a typical batch sorption experiment. Each pressure spike corresponds to dosing 3.8 bar NH_3_ into the batch reactor. The sharp negative slopes following each peak imply that the reaction between ammonia and PA is extremely rapid, consistent with several observations in the literature.^63–65^ Uptake of NH_3_ per dose (*n*_NH_3_, dose_) is calculated using eq 6:

$$n_{NH_{3},\mathrm{dose}}=\frac{\Delta P_{\mathrm{start}-\mathrm{finish}}V_{\mathrm{vapor}}}{RT}$$ 6

Here, Δ*P*_start–finish_ is the pressure differential between the beginning and end of each dose, *V*_vapor_ is the volume of the batch reactor not occupied by the sorbent, *R* is the ideal gas constant, and *T* is the ambient temperature (24 °C). Figure 2 reveals that ammonia uptake (and thus Δ*P*_start–finish_) decreases as time (and the amount of NH_3_ already taken up by the sorbent) increases. We calculate a cumulative absorbed NH_3_:P, or simply N:P, molar ratio of 1.45 by the end of the experiment, demonstrating significant uptake of NH_3_ by PA under these mild conditions that mimics a nearly stoichiometric mixture of MAP and DAP.

**Figure 2:**
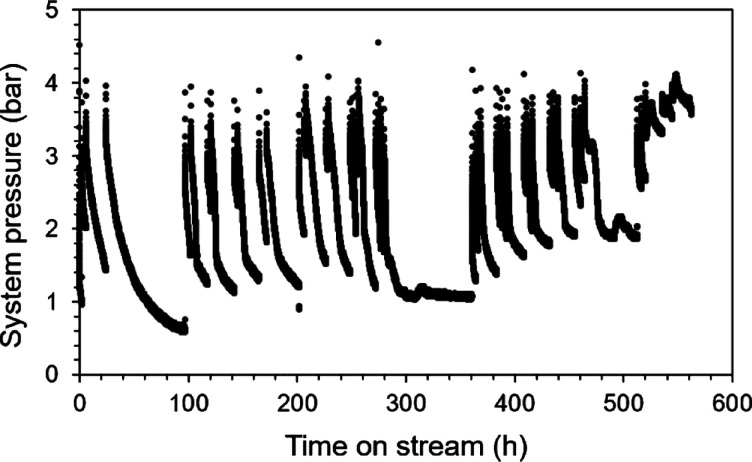
Uptake of repeated (48) doses of 3.8 bar NH_3_ by anhydrous H_3_PO_4_ (13.8 g crystalline H_3_PO_4_ + 0.5 g H_4_P_2_O_7_) at ambient temperature in a glass-lined Hastelloy stirred batch reactor initially filled with Ar at ambient pressure.

However, since Δ*P*_start–finish_ is near, but not exactly, zero at the conclusion of this experiment (0.25 bar), this uptake is not a true reflection of the thermodynamic equilibrium between NH_3_ and ammonium phosphate sorbents under these conditions. Additionally, the long duration of this experiment makes repetitions of it infeasible. This prompted us to invert our approach, instead interrogating the thermodynamic relationships of these systems via their *desorption* reactions (i.e., eqs 2–3, reverse reactions).

### 3.3 Cyclability of Ammonia Sorption

The envisioned use case for NH_3_ sorption involves repeated cycles of its absorption and desorption on a time scale compatible with a continuous, integrated reaction–separation process, preferably on the order of seconds or minutes. Thus, phosphate salts must be utilized at conditions under which both absorption and desorption steps are relatively rapid. To assess this, we ramp the temperature up (desorption) and then down (absorption) in a batch reactor charged with pure DAP in intervals of 50 °C, holding each intermediate temperature for at least 1 h to ensure equilibration. Figure 3 illustrates the results of this cyclic experiment, with NH_3_ release quantified using eq 6, after correcting absolute pressure for ideal gas thermal expansion of Ar originally present in the stirred vessel. Notably, very little NH_3_ is released below 150 °C during desorption, while the absorption step shows that considerable amounts of ammonia (2–4%) are not re-absorbed at or below 150 °C.

**Figure 3:**
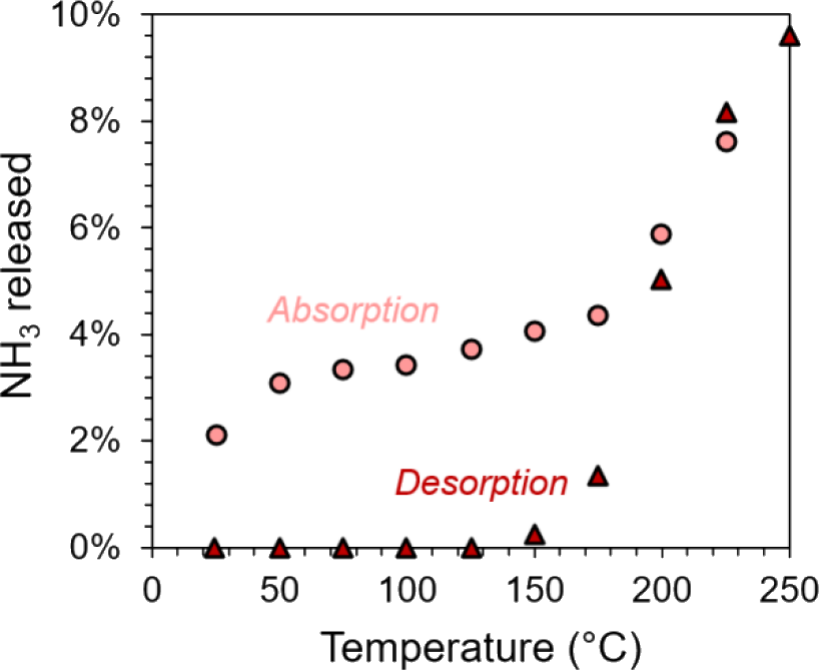
Desorption (dark red triangles) and re-absorption (light red circles) of NH_3_ from/to DAP (amount in mol % relative to total NH_3_ content in DAP) in a stirred batch reactor. Each data point was collected after at least 1 h to ensure pressure and temperature equilibration at the condition.

We hypothesize that this observation is a direct result of mass transfer limitations. Although the phosphate salts are fed as powders, the tabulated melting point^47^ of MAP is 190 °C; the salts transition to molten liquids above this temperature. Upon cooling below 150 °C, the entire contents of the batch reactor freeze into a single solid mass. This mass expectedly has a much lower specific surface area than the original solid powder and cannot be stirred in its frozen state. The lower interfacial area exposed by this re-frozen liquid compared to the original powder necessarily restricts meaningful mass transfer rates and introduces operability challenges for a conceptual sorption process. Therefore, absorption/desorption cycles of NH_3_ in phosphates must be conducted above approximately 200 °C.

Above this threshold, phosphate salts can be used repeatedly in sorption cycles. Pressure and temperature trends (Figure 4a) are shown alongside amounts of ammonia desorbed at the upper and lower temperature points of each cycle (Figure 4b) during repeated thermal cycling of DAP between 200 and 250 °C. Figure 4a confirms that each condition was held for at least 0.75 h to allow for thermodynamic equilibration. Figure 4b illustrates the good reproducibility in the amounts of NH_3_ desorbed between thermal cycles. Specifically, the amount of NH_3_ desorbed at 250 °C varies by less than 1.5% between cycles (10.9–11.8% at 250 °C and 7.7–9.1% at 200 °C). Commercial application would likely require sorbents to be used for thousands of cycles or more with minimal side reactions; the proof-of-concept data depicted in Figure 4 demonstrate that ammonium phosphate systems hold promise to meet this requirement.

**Figure 4:**
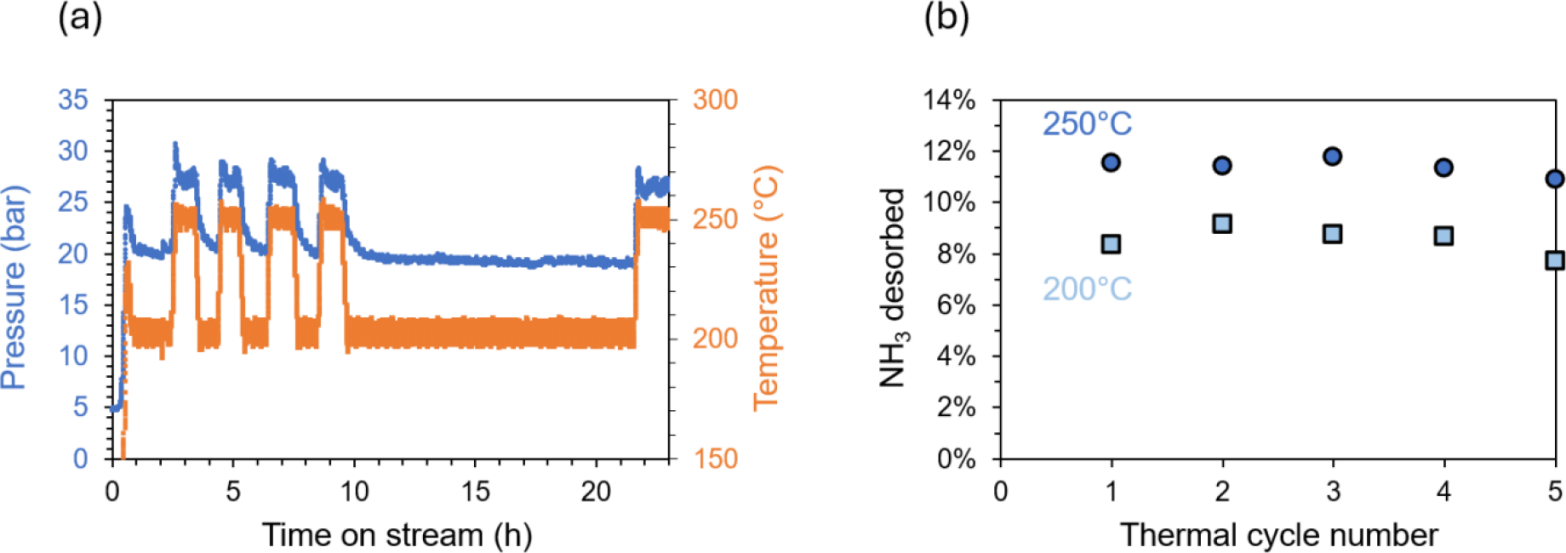
(a) Pressure (blue, left axis) and temperature (orange, right axis) trends and (b) amount of NH_3_ desorbed (mol %) from DAP at 200 °C (light blue squares) and 250 °C (dark blue circles) per cycle during thermal cycling in a stirred batch vessel under an Ar atmosphere.

### 3.4 Composition–Temperature Relationships

Next, we sought to understand sorption behaviors as a function of initial stoichiometry and operating temperature. We measure equilibria between NH_3_ and its two ammonium phosphates by charging batch reactors with physical mixtures of MAP and DAP at defined atomic ratios of nitrogen and phosphorus (i.e., N:P ratios). These stoichiometric ratios are defined according to eq 7:

$$\mathrm{N:P}=\frac{n_{\mathrm{MAP}}+2n_{\mathrm{DAP}}}{n_{\mathrm{MAP}}+n_{\mathrm{DAP}}}$$ 7

These mixed charges of MAP and DAP are then subjected to ramped desorption experiments from 25 to 250 °C (Figure 5). Two intuitive trends are evident from the data. First, increases in the temperature result in additional NH_3_ release from all mixtures. Indeed, appreciable amounts of NH_3_ are only evolved at or above 150 °C. This corroborates our thermodynamic intuition, as the contribution of entropy via the creation of a mole of gaseous NH_3_ (eqs 2–3, reverse reactions) becomes more pronounced with increasing temperature. Second, substances that possess more NH_3_ content in their composition more readily release NH_3_ (i.e., higher *P*_NH_3__ traces in Figure 5). This trend matches the TGA data shown in Figure 1, confirming that higher temperatures are required to desorb NH_3_ from MAP and, equivalently, the second NH_3_ group from DAP compared to its first. To the best of our knowledge, phase diagrams of pure MAP and DAP components have not been reported in the literature, although one MAP–DAP–water ternary phase diagram has been reported.^66^ Elsewhere, one P_2_O_5_–H_2_O phase diagram has been reported.^37^ We emphasize the importance of determining the pure component and solid–liquid–gas phase diagrams of PA, MAP, and DAP in the absence of water to further corroborate our composition–temperature relations for future investigations.

**Figure 5:**
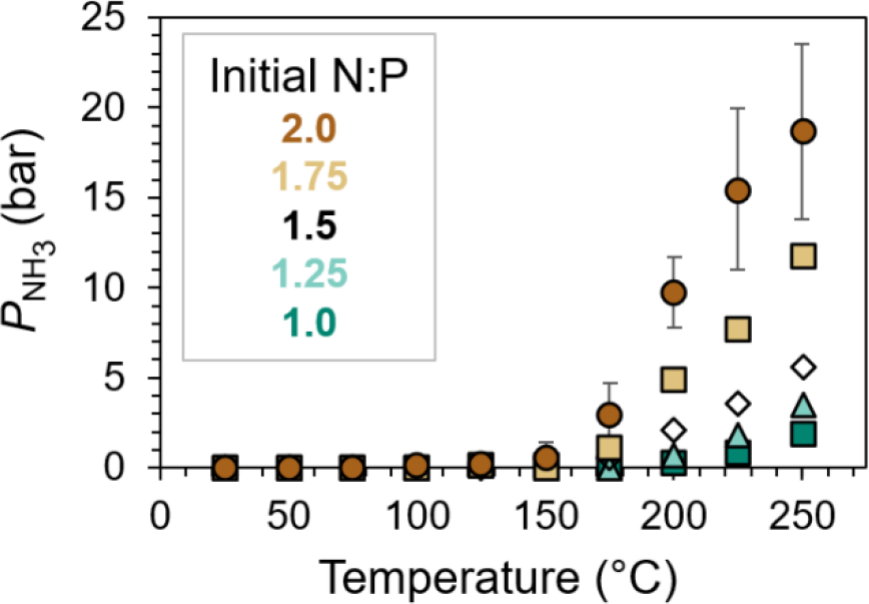
Experimental observations (shapes) and model fits (dashed lines; *vide infra*) of liberated NH_3_ pressures equilibrated with mixtures of MAP and DAP in a stirred batch reactor vessel. Initial N:P varies between 1.0 (pure MAP) and 2.0 (pure DAP); see eq 7. Error bars on N:P = 2.0 samples represent single standard deviations of results obtained from five independent trials.

Seeking other complementary insights into phosphate composition, we attempted a battery of analyses to identify the relative contributions of MAP and DAP in solids recovered following batch sorption experiments. To our frustration, we found that no technique allows for definitive, reproducible solids identification. While ^31^P NMR yields clean, unique signatures for phosphate standards when analyzed in isolation (Figure S3), attempts to calibrate ^31^P chemical shifts to pure phosphate standards were unsuccessful when applied to known mixtures of phosphate salts prepared at N:P ratios of 0 to 2 (Figure S4). We hypothesize that slight variations in p*K*_a_, spin state and/or hydrogen bonding networks that accompany local aqueous and deuterated solvation environments of phosphate mixtures may underlie these confounding observations.

Separately, we pursued ICP-OES analysis for phosphorus content in parallel with CHN analysis for nitrogen quantification (calculating oxygen by difference). Here, uncertainties in each experimental measurement likely compound in such a way that calculated compositions do not sufficiently align with theoretical compositions of each pure and physically mixed standard for elements other than N (Table S1), implying an unsuitable approach. While we observe some experimental agreement in the N:P ratio of 1.45 via gas uptake with the analytical results yielding ∼1.3–1.4 N groups in post-desorption solids, we cannot corroborate these results by the measured P, H, or O content. We further attempted pXRD measurements as another means of discerning relative MAP and DAP content. Encouragingly, some diffractogram peaks are unique to each pure compound (Figures S5–S6), allowing for semi-quantitative empirical calibrations (Figure S7) for fingerprinting via a modified Rietveld refinement approach.^67^ However, we observe peak broadening and angle shifting of maxima in each known mixture and experimental solid (Figures S6–S7) that suggest poor crystallinity and significant disorder among these mixed phases, obscuring the reliable interpretation of their diffractograms. We identify the compositional analysis of mixed ammonium phosphates as an important risk that requires mitigation in follow-up investigations.

Looking forward, other analytical techniques, such as Raman or infrared (IR) spectroscopies and creative *in situ* probes, may help elucidate phosphate mixture compositions. Early into our experimental efforts, we attempted to sample phosphate liquids directly from hot batch reactors, although we quickly learned that these liquid salts readily freeze along liquid sampling tube walls, preventing their collection as aliquots. Another possible characterization approach would be to conduct high-temperature NMR experiments using 1D and 2D (COSY, DEPT-135, HSQC) techniques for ^31^P and ^15^N nuclei; *operando* sorption and desorption events could occur within a specialized NMR tube designed for elevated temperatures and pressures. These and other potential mitigation strategies may satisfy compositional measurements beyond the techniques reported here.

### 3.5 Thermodynamic Model Validation

We formulate a thermodynamic model for sorption equilibria between NH_3_ and its phosphate salts using the data of Figure 5 to enable predictive understandings. The model considers equilibrium of NH_3_ between two phases: vapor-phase NH_3_ (ideal gas) and NH_3_ in a liquid phase consisting of ammonium phosphate salts with any physically possible ratio of N:P ((NH_4_)*_*x*_* H_3–*x*_PO_4_, 0 < *x* ≤ 2). (Theoretically, values of *x* may be as high as 3, resulting in the formation of triammonium phosphate ((NH_4_)_3_PO_4_, TAP) at this limit; however, TAP decomposes to DAP and its secondary products near 70 °C^47^ and is not expected to be favored as a stable product at the conditions of interest to this conceptual process, so we practically consider DAP to be the terminal equilibrium product.) This equilibrium implies equivalent Gibbs free energies of the liquid (*G*_liquid_) and vapor (*G*_vapor_) phases. Assuming that the vapor phase is either pure NH_3_ or only mixed with an inert component (Ar in our hands), we can express the vapor Gibbs contribution as the relationship in eq 8:

$$G_{\mathrm{vapor}}\left(T,P_{NH_{3}}\right)=G_{NH_{3}}^{0}\left(T\right)+RT\ln\left(P_{{\mathrm{NH}}_{3}}\right)$$ 8

Here, *G*_NH_3__^0^(*T*) is the tabulated^68^ reference state Gibbs free energy of NH_3_ vapor, *T* is the absolute temperature (K), and *P*_NH_3__ is expressed in bar. Thus, *G*_vapor_ may be directly calculated for every data point in Figure 5 based on the *T* and *P*_NH_3__ of each measured condition, and each value of *G*_vapor_ may be set equal to an equivalent expression for *G*_liquid_. Our observations in Section 3.4 suggest that *G*_liquid_ depends on N:P and *T*, so we assume a model for this quantity with linear dependencies on each process variable, as described by eq 9:

$$G_{\mathrm{liquid}}\left(\mathrm{N:P},T\right)=A_{0}+{A_{1}}^{*}\left(\mathrm{N:P}\right)+{A_{2}}^{*}T$$ 9

Parametric fits are performed by minimizing absolute residuals to estimate values for *A*_0_, *A*_1_ and *A*_2_ using the 25 desorption data points collected at and above 150 °C and plotted in Figure 5. The root mean squared error of the fit was low (1.247), and the values and 95% confidence intervals for each parameter are reported alongside the best-fit values in Table 1. These parameters may be used in eq 9 to determine *G*_liquid_. Setting this equal to *G*_vapor_ at thermodynamic equilibrium, we may predict an extent of reaction (ξ), the number of moles of NH_3_ released by any mixture of PA, MAP, and DAP, a relation described by eq 10:

$$A_{0}+A_{1}\frac{n_{\mathrm{MAP},0}+2n_{\mathrm{DAP},0}-\xi }{n_{\mathrm{MAP},0}+n_{\mathrm{DAP},0}}+A_{2}T=G_{NH_{3}}^{0}\left(T\right)+RT\ln\left(\frac{\xi RT}{V_{\mathrm{reactor}}}\right)$$ 10

Here, *n*_MAP,0_ and *n*_DAP,0_ are the moles of MAP and DAP, respectively, present in the batch reactor before heating, *V*_reactor_ is the volume of the system, and $\frac{\xi RT}{V_{\mathrm{reactor}}}$ is the pressure of NH_3_ in the reactor predicted by the ideal gas law.

**Table 1:** Best-Fit Parameter Values and 95% Confidence Interval Bounds for Liquid Ammonium Phosphate Gibbs Free Energy Parameters^a^

parameter^b^	best-fit value^a^	lower bound^a^	upper bound^a^	units^b^
*A*_0_	–141	–149	–132	kJ mol^–1^
*A*_1_	13	11	14	kJ mol^–1^(N:P)^−1^
*A*_2_	0.27	0.25	0.28	kJ mol^–1^ K^–1^

a Parametric fits and statistical analysis performed using MATLAB R2022a Curve Fitting Toolbox by minimizing absolute residuals for parameters *A*_0_, *A*_1_ and *A*_2_ of eq 9 using the ≥150 °C desorption data visualized in Figure 5.

b See eq 9 and surrounding discussion in main text.

The thermodynamic relationship described by eq 10 may be conveniently used to predict ξ, and therefore *P*_NH_3__, for any set of reactor contents and temperature. We performed this exercise for each point in our data set to compare the predicted and observed NH_3_ pressures (Figure 6a) and to generate a lag plot of residuals (Figure 6b), defined as the difference between observed and predicted NH_3_ vapor pressures. All points in Figure 6a are close to *y* = *x*, illustrating the goodness of model fit in describing the experimental data. Furthermore, the lag plot (Figure 6b) exhibits randomly distributed points, confirming that the model has no major systematically correlated errors. Model predictions are plotted as dashed lines in Figure 5. Taken together, these plots demonstrate that the model of eq 10 accurately captures the data, serving as a useful guide for the reaction engineering of equilibrium-staged reactive sorption units.

**Figure 6:**
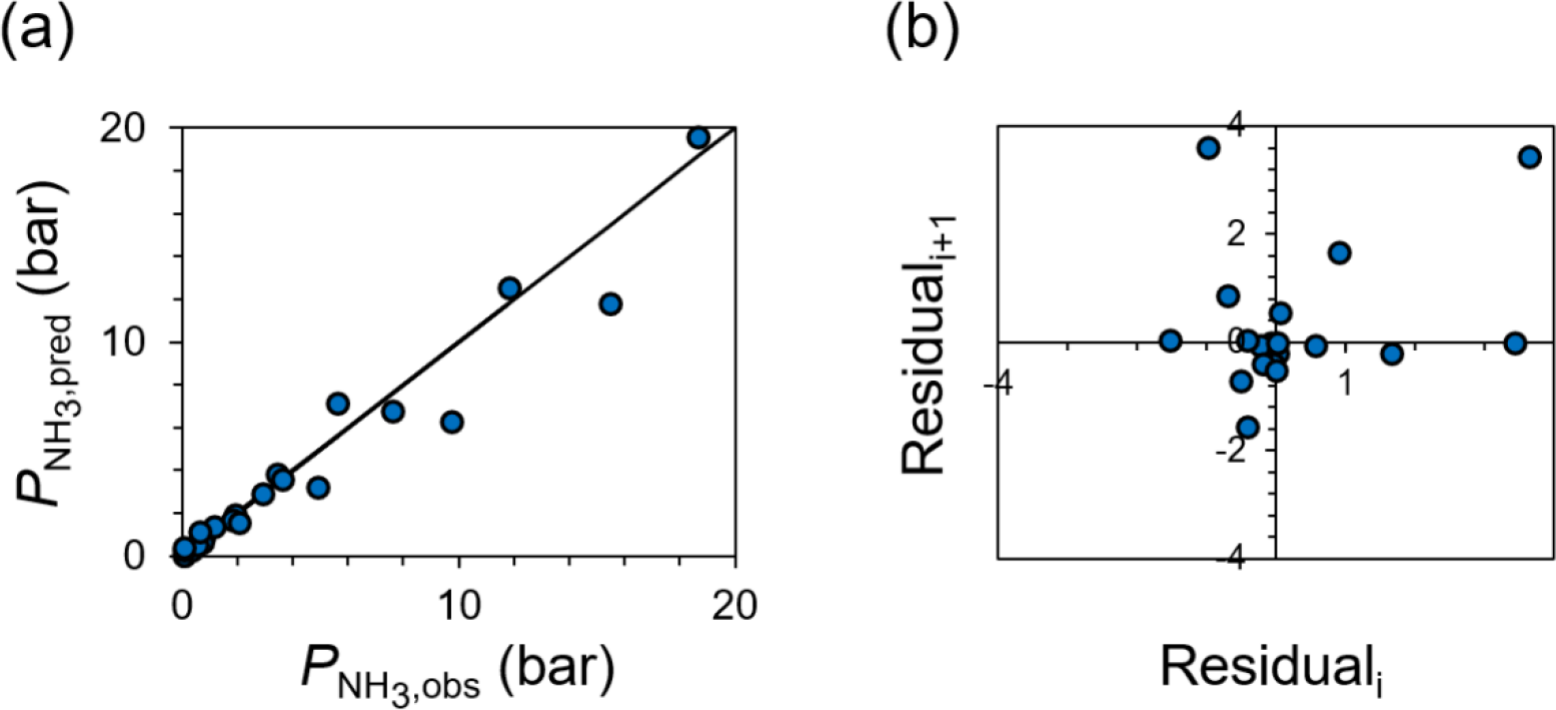
(a) Predicted and observed NH_3_ vapor pressures, with *y* = *x* included as a solid line, and (b) a lag plot of residuals of the ammonia–ammonium phosphate equilibrium model described by eq 10. See derivation and discussion in the main text.

### 3.6 Molecular Modeling

We investigated the molecular dynamics (MD) of a binary NH_3_–H_3_PO_4_ system with the goal of computing *P*_NH_3__ at relevant N:P stoichiometries to corroborate our thermodynamic understanding of this ammonia sorption approach. However, classical MD simulations cannot handle reactions between NH_3_ and PA/phosphate ions (eqs 2–3) due to the harmonic model of chemical bonds used in force fields. Instead, we attempt AIMD based on each DFT and ReaxFF MD simulations^48–54^ to investigate predictive trends in *P*_NH_3__ for the NH_3_–H_3_PO_4_ system at different N:P stoichiometries. We immediately learned that DFT predictions of ammonia vapor pressure appear overestimated (Figure 7a, N:P = 2, dotted black line) relative to experimental measurements (dashed black line)—implying that DFT either cannot adequately capture the electrostatics and hydrogen bonding behaviors of this highly polar molecular system or that it may need a much longer simulation time to reach equilibrium than the 25.0 ps used here. However, due to the very high computational cost of DFT calculations, it is infeasible to run longer simulations; further, for N:P ≤ 1, virtually no NH_3_ molecules are present in the gas phase, leading to zero vapor pressure.

**Figure 7:**
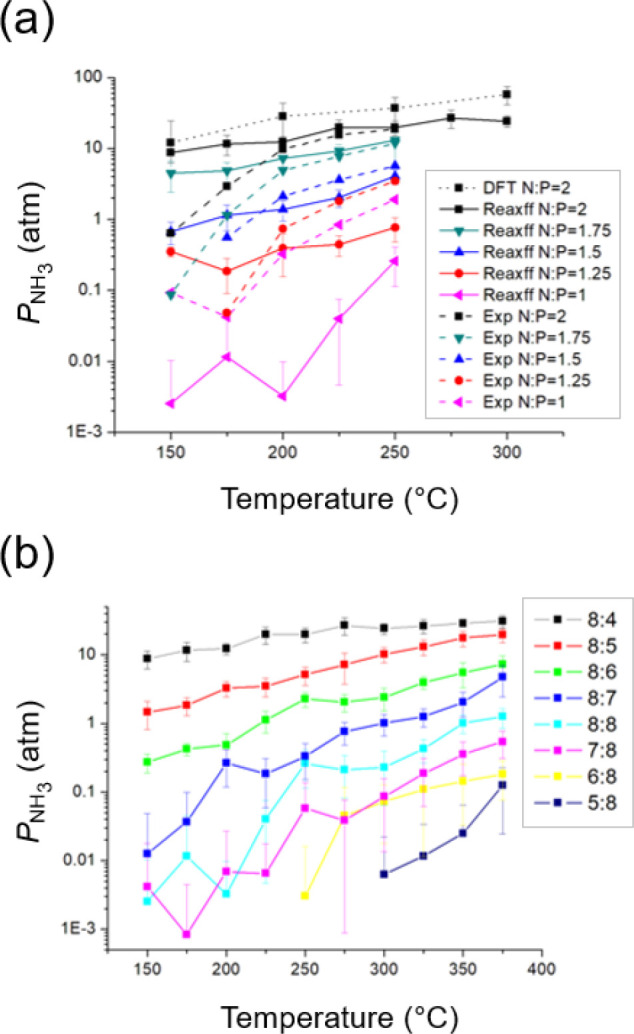
(a) Ammonia vapor pressure (atm) predicted from AIMD (DFT, dotted black line), ReaxFF MD (Reaxff, solid lines), and experimental observations (Exp., dashed lines; see Figure 5) at various temperatures and for initial N:P ratios spanning 1–2 (shapes). (b) Ammonia vapor pressure (atm) predicted at different temperatures and N:P ratios, expressed as NH_3_:H_3_PO_4_ molecular ratios. Error bars represent a single standard deviation over at least 10 trials.

Seeking an alternate approach, we custom-built a reactive force field (ReaxFF) that allows for much longer simulation times (3.0 ns) to enable meaningful vapor pressure predictions across a range of NH_3_:H_3_PO_4_ ratios (8:4 to 5:8) for system temperatures spanning 150–375 °C (Figure 7b). Overall, model predictions exhibit good agreement with experimental data between 200 and 250 °C and for N:P ≥ 1.5 while closely following the trend of DFT AIMD simulation results (Figure 7a). The underestimation of computational predictions at low temperatures and stoichiometries may be due to liquid–solid phase transition phenomena that are inadequately captured by ReaxFF MD; deliberate phase transition models require a more accurate force field and significantly longer simulation times, or else multistage MD frameworks where reactions and non-reactive phase transitions are compartmentalized in coupled model tiers solved simultaneously to predict NH_3_ vapor pressure and other properties. However, employing either approach to capture phase change phenomena is beyond the scope of this research. Indeed, we do not observe any crystallization in these simulation sets, and the condensed phase remaining as liquid is also confirmed by a stable diffusion coefficient for H_3_PO_4_ and phosphate ion species during simulation. We also affirm that H_3_PO_4_ is predicted to have a negligible vapor pressure. Nonetheless, the observed agreement aligns well with target operating conditions (Figure 4 and the surrounding discussion), further corroborating our macroscopic understanding and tuning of the equilibrated MAP and DAP mixture as a functional sorbent.

Finally, customized ReaxFF frameworks offer unique practical insights into physical phenomena that are otherwise difficult or inaccessible to measure by experiment. One such physical parameter is the dimensionless group for Henry’s law constant (*H*_A_ = *C*_A, dissolved_/*C*_A, gas_), which describes the proportionality of dissolved gas “A” in a liquid to its free concentration above the liquid. ReaxFF enables direct estimation of Henry’s law constants across a range of industrially relevant operating temperatures (Table 2), crucially while accounting for its reactivity with the liquid medium. We hypothesize that the distinct temperature dependencies of ammonia solubility, ammonia partial pressure and its reactivity with the sorbent result in a non-monotonic trend of Henry’s law constants with increasing temperature. Considerable quantum chemical error is noted in the 200–240 °C range where many experiments were conducted and where solute/solvent reactivity is very rapid, implying extended coupling of these phenomena in ReaxFF MD. Nonetheless, insights into Henry’s law coefficients offer additional practical utility to reaction engineering and process design specifications.

**Table 2:** Henry’s Law Constants^a^ for NH_3_ Solute in H_3_PO_4_ Reactive Solvent Predicted by ReaxFF MD Simulations

**temperature (°C)**	**Henry’s law constant** ^a^ **(dimensionless)**
180	24 ± 7
200	25 ± 11
220	33 ± 11
240	40 ± 18
260	24 ± 10
280	30 ± 10
300	17 ± 6
320	23 ± 7
340	25 ± 9
360	16 ± 4
380	16 ± 3

a Estimated from 3 ns ReaxFF simulations. See the SI and main text for details. The second value represents a single standard deviation computed across ten unique trials.

### 3.7 Process Modeling and Technoeconomic Analysis

Finally, we benchmark our liquid sorption approach with conventional condensation separation methods within an otherwise traditional Haber–Bosch synthesis plant using the Aspen Plus process simulator. The nominal ammonia production rate is 100 tonnes d^–1^, and production of N_2_ and H_2_ synthesis gas components is not included in the system boundary to keep ammonia synthesis loops and the variable downstream separation trains (i.e., condensation vs liquid sorption) disconnected from upstream feedstock processing. Because of this key flowsheet constraint, there are many potential heat integration and mass recuperation routes available depending on the sources of, and routes to, reactants N_2_ and H_2_. To this end, we acknowledge that this is a preliminary process design that is not optimized to reduce utility consumption or capital costs; instead, process models intended for direct comparison are designed using appropriate assumptions and caveats to mimic the experimental data where possible and to identify potential operability hurdles. Complete details on process model development and technoeconomic analysis (TEA) are reported in a comprehensive SI discussion alongside Figures S8–S11, Tables S2–S8, and eqs S1–S11.

Briefly, ammonia synthesis is carried out at either 300 or 150 bar in three adiabatic reactor beds arranged in series with interstage cooling (Figure S8); fresh stoichiometric feed (eq 1) is injected before each reactor stage (Table S2), with the first preheated to 370 °C. The final reactor stage’s effluent stream exchanges heat with the feed gas before it enters one of the two separation trains: (i) a conventional condensation section (Figure S8) or (ii) a phosphoric acid absorption and DAP desorption loop (Figure S9). For the latter, the effluent of the synthesis train is fed to an isothermal absorber operating at 200 °C and 145 bar, represented here as a stirred tank reactor followed by an absorption column in series. The stirred tank reactor is used as a pre-neutralizer to enforce uniform mixing and temperature control for the first reaction of ammonia with PA, pushed to complete conversion to DAP in the subsequent absorption column. Then, the DAP-rich stream is sent to a ten-stage desorption column operating at 250 °C and 18.7 bar to liberate the NH_3_ product and to regenerate PA. The exotherms released from the absorption stirred tank and column are integrated with the reboiler of the desorption column. Complete material and energy balances are included in Tables S5–S6. All equipment in the sorption section is assumed to be made of Hastelloy to mitigate corrosion. Each process combination was simulated for the upstream synthesis scenarios to estimate the performance and cost.

We then compare preliminary economic projections using a centralized production model^55^ modified for ammonia production as a discounted cash flow model, while capital costs are estimated using a bare-module approach for ten unit operations: the three-bed reactor, three coolers, two heat exchangers, two compressors, a flash vessel for condensation and a cooling tower. Results of bare-module capital expenditures (2022 USD) are described for the conventional process (Table S7) and liquid sorption (Table S8). Operating expenditures primarily consist of feedstock costs, utility, labor and maintenance costs. Feedstock costs are fixed at 0.05 $ kg^–1^ N_2_, 2.00 $ kg^–1^ H_2_, and 0.00011335 $ gal^–1^ cooling water, while utility costs (primarily electricity for compression) are 50 $ MWh^–1^. Other operating and maintenance (O&M) assumptions are described in the SI.

Taken together, these analyses allow for preliminary TEA of levelized ammonia production costs for a side-by-side comparison of ammonia separation approaches integrated into otherwise identical synthesis loops agnostic to their N_2_ and H_2_ sources. The production cost of ammonia in the conventional process is estimated to be 567 $ t^–1^ NH_3_ (Figure 8a), compared to the higher price estimate of 866 $ t^–1^ NH_3_ resulting from the liquid sorption-based process (Figure 8b). As expected, hydrogen prices constitute 78% of the ammonia production cost and 89% of the feedstock costs in the conventional scenario. Sensitivity analyses depicted as tornado plots (Figures S10–S11) further corroborate that the most cost-sensitive parameter is the price of hydrogen purchased at the plant gate, which is in part a consequence of the process boundaries defined for this exercise; for the sorption-based case, electricity cost also manifests as a cost-sensitive variable. Feedstock costs increase slightly in the absorption scenario from 446 $ t^–1^ NH_3_ to 473 $ t^–1^ NH_3_ due to the additional closed-loop and makeup PA inputs and increased cooling water usage. Most notably, the utility cost increases from 62 $ t^–1^ to 322 $ t^–1^ in this case, in part due to the high pumping demands required to move large volumes of viscous PA, MAP, and DAP streams (Table S6). We note that the process simulation used for this TEA is deliberately configured to achieve the same ammonia production rate regardless of the separation method; therefore, a main culprit behind increased utility costs in the sorption-based process is the superposition of likely unoptimized process conditions within this segment. Despite this, the sorption process model reasonably emulates experimental observations, offering a useful preliminary comparison with established ammonia condensation recovery methods, although it is clear following our analysis that the incumbent approach remains advantageous.

**Figure 8:**
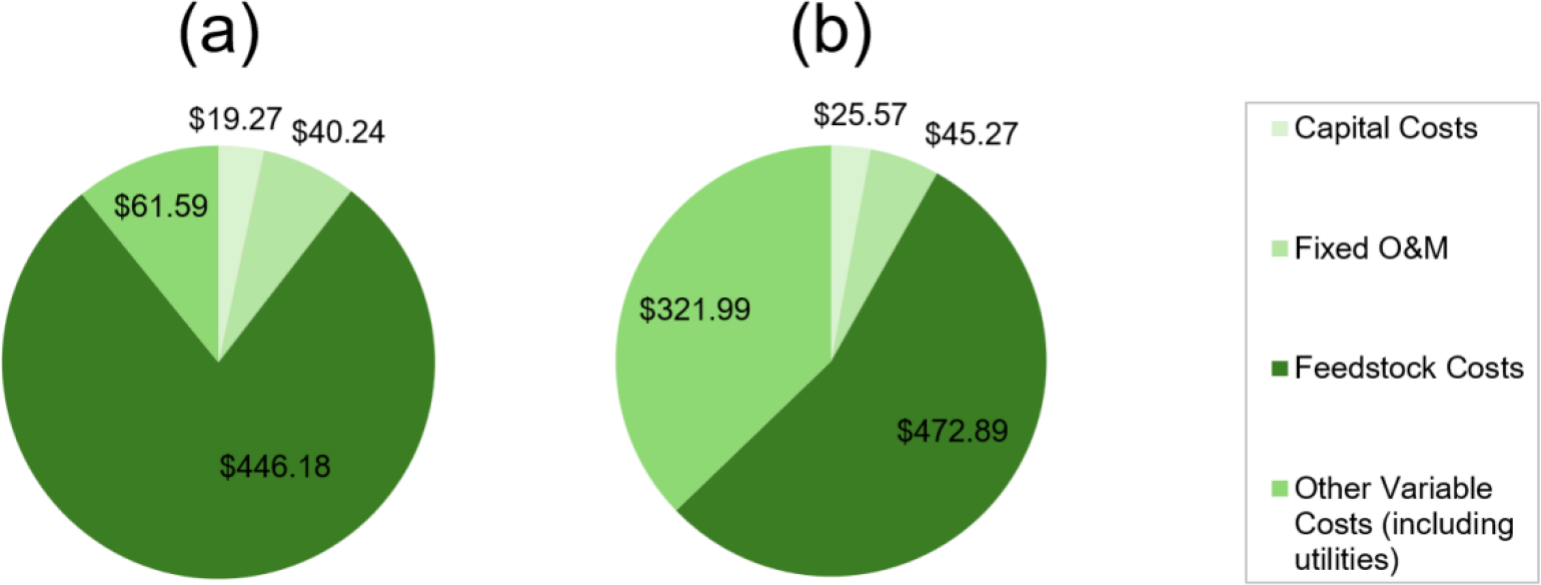
Estimated ammonia production costs via processes involving (a) conventional condensation (567 $ t^–1^ NH_3_) and (b) liquid sorption by phosphoric acid (866 $ t^–1^ NH_3_) for a 150 bar synthesis plant.

Some opportunities to optimize the process simulation are presented next to aid in follow-on investigations. First, the absorber unit could operate at a higher pressure to reduce the pressure swing incurred from compression that hampers the sorption-based process (Figure 8b, Figure S11). Ideally, the absorber would operate at or near the same pressure as the upstream synthesis reactor to enable near-isobaric integration. Either of these scenarios would also likely require higher operating temperatures to achieve an equivalent desorption of ammonia gas, potentially negating some of the compression energy savings. Otherwise, reducing the utility costs from compression could be achieve in two ways: (i) improving desorption ammonia release at elevated temperatures and pressures to reduce the pressure difference between sorption units, thus reducing the pumping costs; or (ii) operating an even greater temperatures to volatilize H_3_PO_4_ so as to reduce liquid flow rates, and thus pumping demands. Unfortunately, both scenarios extend well beyond the reaction conditions studied in this paper, and without proper experimental validation, their thermodynamic behaviors cannot be extrapolated to the process models presented above to thoroughly evaluate their efficiency and economic consequences, instead posing them for future studies.

In an ideal scenario, retrofitting a Haber–Bosch plant with an ammonia sorption system would offset any modest increase in capital costs with reduced utility usage from reduced compression requirements. Modeling and validating higher-pressure absorption units to further reduce recompression costs could offer potential advantages along this vein, although the impacts may be more modest than originally hypothesized. The intrinsically high selectivity of liquid absorption allows for a readily pure ammonia gas product to be released during the desorption step; hence, further savings may be realized by obviating downstream refrigeration cycles, although these scenarios extend beyond the boundaries of our system analysis. Other advantageous economics may be unlocked by valorizing MAP or DAP slipstreams as common, fungible fertilizer coproducts.

Overall, our simplifying assumptions may be revisited in light of expanded, refined vapor–liquid equilibria, gas solubility measurements, and other physicochemical properties that could collectively inform the development of a rigorous electrolyte-based property model in Aspen Plus, allowing for targeted process integration and thus more definitive TEA conclusions. We anticipate that additional savings may exist beyond the above limited scenarios and segmented analyses if synthesis reactors could realistically operate close to absorption pressures and vice versa, offering potential synergies or even process intensification with lower-pressure synthesis routes for next-generation ammonia production.

## 4 Discussion

The fundamental thermodynamic insights and potential process advantages afforded by ammonia liquid sorption enable practical reaction engineering advances alongside straightforward unit operation design criteria for this equilibrium-based reactive separation. The proof-of-concept information reported here establishes early viability of this promising approach with a defined path toward process development and de-risking. In this section, we highlight several key advantages and potential drawbacks of ammonia liquid sorption by phosphate salts.

Despite its potential as a liquid sorbent for NH_3_, phosphoric acid possesses some unfavorable qualities. As a trivalent acid, proton p*K*_a_ values span a large range (2.1, 7.2, and 12.3), and the corrosivity of anhydrous PA may shorten the serviceable lifetimes of vessels, piping and other wetted materials of construction. The service life of the sorbent itself is expected to be a strong function of commercial purity, corrosion (e.g., leached metal accumulation), materials compatibility (e.g., degraded organic component accumulation from wetted seals), overall throughput, and other operability properties. Exceeding the melting points of PA, MAP, and DAP would require extensive heat tracing in process lines to avoid freezing. As non-aqueous working fluids, moderate to high viscosities may require specialized pumps and other non-standard handling protocols, although ammonium phosphate slurry viscosities are known to decrease with increasing temperature.^69^ Multiscale modeling may elucidate a better understanding of hydrodynamic challenges and phase transitions beyond the reach of DFT and ReaxFF MD tools. Special attention to developing compatible sorbent and material sets, particularly with respect to corrosivity at elevated temperatures, is urged to facilitate meaningful reduction to practice.

An additional challenge of H_3_PO_4_-based NH_3_ sorption approaches is the extremely fast reaction kinetics, observed both in our hands (Figure 2) and by several others.^63–65^ Instantaneous exothermic reactions such as acid–base neutralizations pose operability challenges (e.g., mixing limitations, thermal gradients) and safety hazards (e.g., hot spot formation and thermal runaway) that hamper implementation. While kinetics is not expected to be performance-limiting, inter- and intraphase mass and heat transfer may control effective sorption rates, requiring an alternative design basis for equilibrium-staged absorber columns. However, appropriate selection of gas–liquid contacting patterns would mitigate interphase transport limitations and shorten single-phase mixing time scales, enabling thermodynamic control of sorption steps.

To this end, ammonia production processes that avoid pure PA or PA-rich streams altogether may possess inherent advantages. From Figure 4, we infer that a practical approach may be to instead swing between MAP and DAP as the favorable liquid sorbent mixture. The first MAP proton dissociation is shifted approximately 5 p*K*_a_ units higher than that of the first PA proton, alleviating potential corrosivity challenges of handling pure PA (Figure S1). Cycling exclusively between MAP and DAP also avoids the viscosity and crystallization challenges of potential TAP byproduct formation and crystallization under excess ammonia feeds.

Beyond individual unit designs, process integration opportunities exist to leverage the potential advantages of heat and matter flows within commercial settings. Isobaric integration of liquid sorption with Haber–Bosch reactors could be carried out as separate steps (Scheme 1), or it could feature multiple temperature zones to facilitate intensified, *in situ* ammonia product capture, favorably shifting the equilibrium of eq 1 past traditional reactor limits. Established adiabatic reaction engineering strategies such as interstage cooling could conceivably incorporate a “cold” liquid sorbent injected directly between reactor stages to capture a “hot” NH_3_ product, to cool unreacted N_2_ and H_2_ and to reset the equilibrium for the subsequent catalyst bed stage—all while obviating the substantial energy and work required to decompress, condense, recompress and recycle the gaseous reactant streams in conventional Haber–Bosch processes. Critically, the incompressible nature of liquid sorbents intrinsically minimizes the pump work required for sorbent recycle loops (Scheme 1). A fundamental understanding of PA, MAP, and DAP interactions with Haber–Bosch synthesis catalysts and their impacts on catalytic activities (e.g., pore fouling, poisoning, metal leaching, deactivation) is needed to validate and down-select appropriate process intensification concepts.

Intensified synthesis and sorption would further directly lower reactor capital costs from active NH_3_ capture (and thus lower operating pressure) as well as increase throughput per volume due to the associated favorable equilibrium shift (eq 1). Smaller scale or modular systems may be a promising venue for adoption of liquid sorption and the strategies described above, offering more accessible heat management strategies, adaptability to multiple hydrogen/nitrogen sources, and co-location with agricultural communities; in particular, liquid sorption processes afford the coproduction of valuable phosphate fertilizer products alongside ammonia within a single decentralized setting. At any scale of production, the ability to accommodate lower liquid sorption feed pressures further enhances integration with future ammonia production strategies (e.g., electrochemical, photochemical, inductive heating and plasma routes) that leverage alternate reaction paths and/or energy inputs to drive the synthesis of this vital molecule. Ammonia separation challenges and opportunities will no doubt accompany the new pathways.

Accordingly, reaction engineers have several future options to evaluate for NH_3_ sorption vessel configurations, ranging from bubble columns (open, staged, multishaft, packed, and/or slurry) and trickle beds to heat-exchanger-integrated polytropic or loop reactors, among others. Reactor selection will ultimately hinge on the ability to minimize NH_3_ mass transfer resistance and/or hydrodynamic limitations that inhibit the approaches to equilibrium in each eq 1 and eqs 2–3; the fundamental information that underlies Figure 5, eq 10, and Tables 1–2 provides insights into key single-equilibrium-stage behaviors, offering guideposts for multistage unit operation design and integrated process innovations. Myriad sorbent/reaction fluid compositions, fluid–solid contacting patterns, and process intensification approaches exist and are worthy of future investigation.

## 5 Conclusions

In this study, we investigate the liquid absorption and desorption of NH_3_ by charging synthetic phosphate sorbent mixtures in batch reactors, generating the first reported thermodynamic relationships among ammonia and its phosphate salts that are validated by experimental data and molecular dynamics theory. These fundamental insights afford practical calculation bases for reaction engineers seeking to design continuous multistage, equilibrium-based unit operations that could execute this reactive sorption strategy in industrial settings.

Leveraging these knowledge sets, we develop an elementary flowsheet to benchmark our conceptual liquid sorption-enhanced process to the existing ammonia condensation trains that follow Haber–Bosch synthesis loops. Looking forward, we identify key cost drivers and reasonable paths to energy savings that collectively motivate future process development required to realize this technology. While not opportune for commercial plant retrofits, ammonia liquid sorption holds early promise for enhancing Haber–Bosch process efficiencies and cost savings, particularly at small scales for emerging modular synthesis approaches that operate at lower pressures.

Overall, the ammonia sorption strategy presented here is an elegant acid–base reactive separation approach that leverages independent liquid and gas phases for facile ammonia recovery from conventional and emerging production processes. Crucially, the incompressible nature of anhydrous phosphoric acid, monoammonium phosphate, and diammonium phosphate—which together function as suitable liquid sorbents for ammonia uptake and release under process-relevant conditions—offers pathways to potentially significant energy and cost advantages relative to incumbent ammonia condensation methods. While the scale of energy consumption in the 111-year-old Haber–Bosch process is daunting, opportunities to mitigate these massive resource sinks and to transition to future chemical process innovations are increasingly within reach.

## Supplementary Material



## Notes

Detailed molecular model development and simulation details; ^31^P NMR spectra, elemental analysis, and pXRD of standards and substrates and (attempted) calibrations for each technique; detailed process modeling, fitted parameters, material and energy balances, technoeconomic methods, assumptions, sensitivity analysis, and discussion (PDF)

## ‡

LanzaJet, Inc., 520 Lake Cook Road, Deerfield, Illinois 60015, United States
