# Supplementary material for: Liquid Sorption-Enhanced Haber–Bosch Process
Source: Ind Eng Chem Res. 2025 Oct 28;64(44):20930–42. doi: 10.1021/acs.iecr.5c02713 (PMC12593348; doi:10.1021/acs.iecr.5c02713)
Supplement: Supplementary file 1 [file ie5c02713_si_001.pdf]

Supporting Information for

Liquid sorption-enhanced Haber-Bosch process

Nicholas E. Thornburg[1†]*, Jacob H. Miller[2†], William Xi[3], Hai Long[4], Kathleen D. Brown[2], Cheyenne Paeper[2], Rianna Martinez[2], Gabrielle A. Kliegle[2], and Bryan S. Pivovar[5]*

[1] Energy Conversion and Storage Systems Center, National Renewable Energy Laboratory, 15013 Denver West Parkway, Golden, Colorado 80401, United States

[2] Catalytic Carbon Transformation and Scale-up Center, National Renewable Energy Laboratory, 15013 Denver West Parkway, Golden, Colorado 80401, United States

[3] Strategic Energy Analysis Center, National Renewable Energy Laboratory, 15013 Denver West Parkway, Golden, Colorado 80401, United States

[4] Computational Science Center, National Renewable Energy Laboratory, 15013 Denver West Parkway, Golden, Colorado 80401, United States

[5] Chemistry and Nanoscience Center, National Renewable Energy Laboratory, 15013 Denver West Parkway, Golden, Colorado 80401, United States

* nicholas.thornburg@nrel.gov (303-275-4885); bryan.pivovar@nrel.gov (303-275-3809)

[†] These authors contributed equally.

Contents

Figure S1. Photographs of Hastelloy batch reactors following extended exposure to H_3PO_4

Detailed computational modeling methods

Figure S2. Diagram of the *x*–*z* cross section of simulation boxes used in computational studies

Figure S3. ^{31}P nuclear magnetic resonance (NMR) spectra of phosphoric acid and ammonium phosphate standards in D_2O

Figure S4. Relationship between N:P ratio and ^{31}P NMR chemical shift

Table S1. Summary of elemental analyses of phosphate standards and post-sorption solids

Figure S5. Powder X-ray diffraction (pXRD) patterns of phosphate standards

Figure S6. pXRD of MAP and DAP physical mixtures

Figure S7. pXRD patterns and attempted calibration of select post-sorption experimental samples

Detailed process modeling and technoeconomic methods, assumptions and analysis

Figure S8. Conventional ammonia synthesis and separation process flowsheet

Figure S9. Sorption-based ammonia separation process segment flowsheet

Table S2. Reactor bed specifications within process models

Table S3. Regressed parameters for temperature-dependent Henry's law function

Table S4. Regressed equilibrium reaction parameters for DAP decomposition

Table S5. Material and energy balances for the 150-bar conventional ammonia production model

Table S6. Material and energy balances for the 150-bar sorption-enhanced ammonia production model

Table S7. Bare-module capital cost estimates of major equipment in the conventional ammonia production process

Table S8. Bare-module capital cost estimates of major equipment in the sorption-enhanced ammonia production process

Figure S10. Tornado sensitivity analysis plot of key cost parameters for the conventional 150-bar ammonia synthesis process

Figure S11. Tornado sensitivity analysis plot of key cost parameters for the sorption-enhanced 150-bar ammonia synthesis process

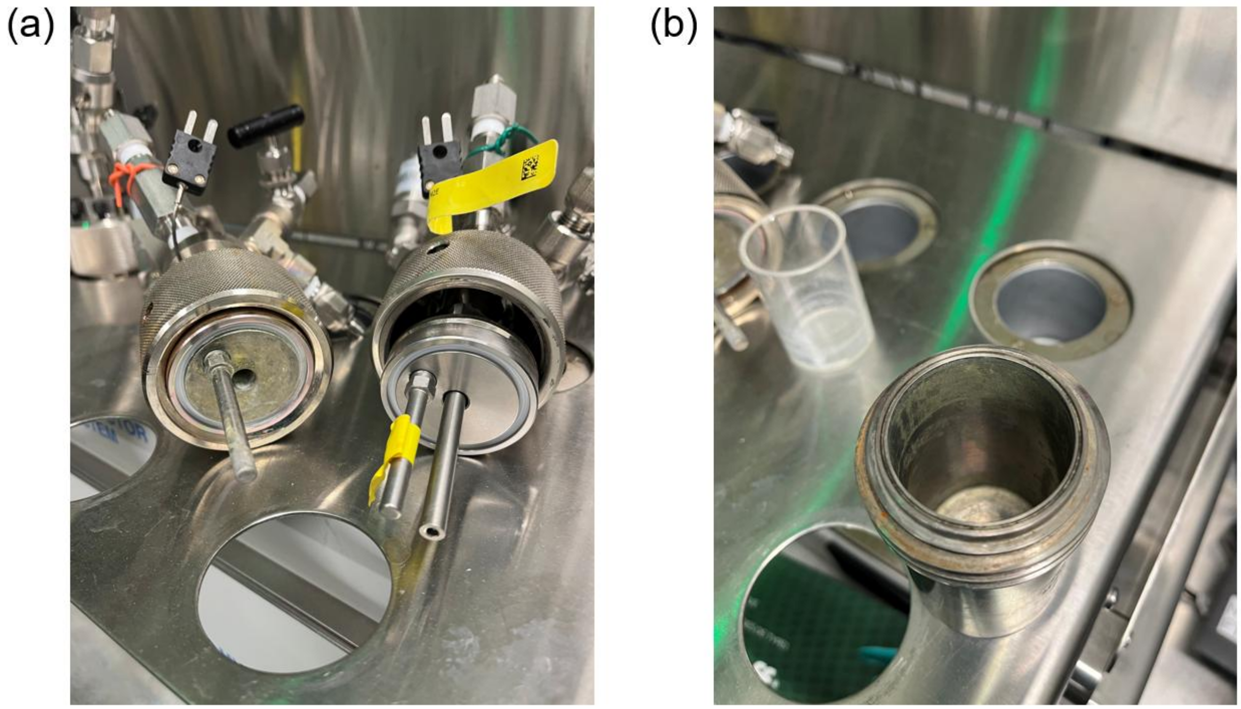

Figure S1. Photographs of Hastelloy batch reactor components. (a) Headpiece wetted internals following extended H_3PO_4 exposure (*left*) next to an unused headpiece (*right*). (b) Vessel body following extended H_3PO_4 exposure. Exposure conditions pertain to experiment described in **Section 2.1**, **Section 3.2** and **Figure 2** of main text. *Photo credit: Kathleen Brown, NREL.*

Complete computational modeling methods

AIMD simulations were performed for a system with 64 NH_3 and 32 PA molecules by the Vienna *ab initio* simulation package (VASP) version 5.4.4[1-3] with periodic boundary conditions in all three dimensions. During the AIMD, the generalized gradient approximation (GGA) Perdew–Burke–Ernzerhof potential for exchange correlation functional was used with a cutoff energy of 500 eV.[4] The system was first equilibrated at 150°C with the isothermal–isobaric (NPT) ensemble for 10 picosecond (ps), resulting in a simulation box of 1.42 nm × 1.42 nm × 1.42 nm. Then, the box's *z*-dimension was increased to 5 nm, leading to a free space for NH_3 to evaporate (**Figure S2**). The enlarged box was equilibrated for another 15 ps with the canonical ensemble (NVT) at 150°C, 200°C, 250°C and 300°C. Finally, for each simulation system, a 25-ps production run was performed. The step size for all AIMD simulations was 1 femtosecond (fs).

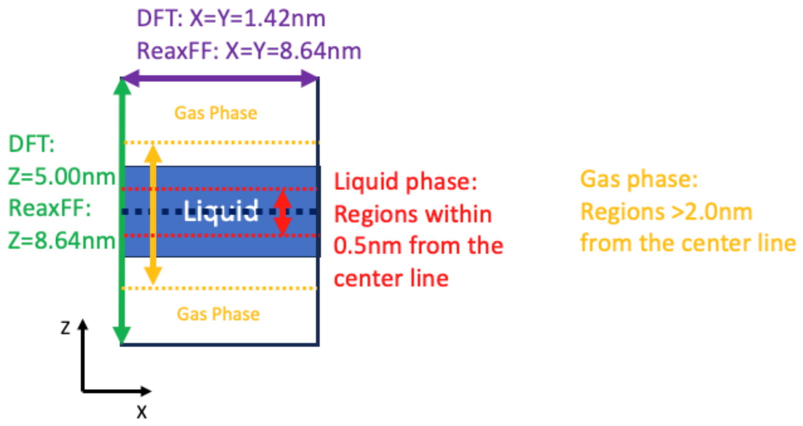

Figure S2. Diagram for the *x*–*z* cross section of simulation boxes used in computational studies.

The ReaxFF model was adapted from Shin et al.[5] for O, H, and P atoms, and Shan et al.[6] for N atoms. In order to prevent unrelated reactions and only focus on **Eqs. 2–3** in the main text between NH_3 and PAs/phosphate ions, parameters for interactions between N–N, O–O, N–O, P–N, and P–H are removed from the force field. Simulations were performed using LAMMPS

package released on Jun 23, 2022[7] with the ReaxFF package and periodic boundary conditions in all three dimensions. The simulations were first prepared by equilibrating 64 NH_3 and 64 PAs at 150°C with NPT ensemble for 1 nanosecond (ns). However, we observed such a small simulation box exhibits poor computational scaling. Therefore, the equilibrated system was replicated 4× in *x*-dimension and 4× in *y*-dimension, respectively, leading to a larger box with 1024 NH_3 and 1024 PAs. This serves as the foundation to build simulation boxes with different ratio of NH_3:PA by randomly removing certain molecules. Then, the *z*-dimension was increased to create a free space for NH_3 to evaporate, leading to simulations boxes of 8.64 nm × 8.64 nm × 8.64 nm (**Figure S2**). Finally, each system was equilibrated for 0.5 ns at different temperatures from 150°C to 375°C with NVT ensemble. The number of NH_3 in the gas phase was monitored to ensure the system reaching equilibrium, and then the final production run lasts for 1 ns for each system. The step size for all ReaxFF MD simulations was 0.5 fs.

In order to exclude the boundary fluctuation near the liquid–gas interface, we define the gas phase and the liquid phase as shown in **Figure S2**: First, we locate the center line of the liquid phase, that is, the *z*-position that has the maximum PA's concentration. Then, the liquid phase is defined as the region within 0.5 nm from the center line, while the gas phase is the region >2 nm from the center line.

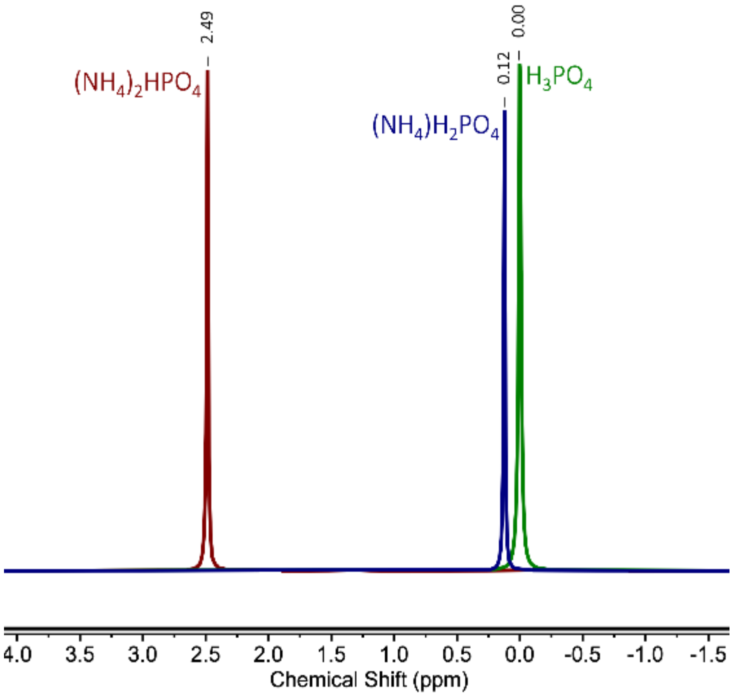

Figure S3. ^{31}P NMR spectra of standards phosphoric acid (H_3PO_4. PA, green), monoammonium phosphate ($(NH_4)H_2PO_4$, MAP, blue) and diammonium phosphate ($(NH_4)_2HPO_4$, DAP, red) analyzed independently in D_2O solvent. Inset number labels indicate ^{31}P chemical shift (ppm).

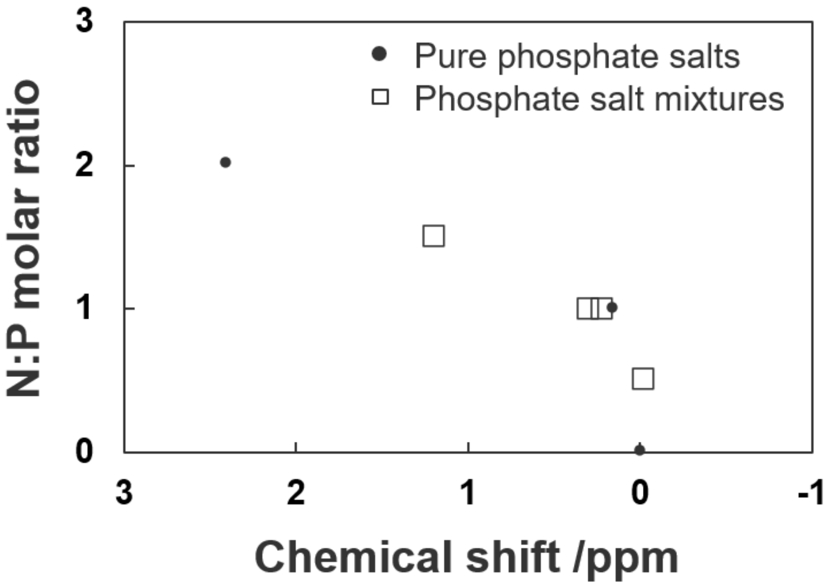

Figure S4. Relationship between N:P molar ratio and ^{31}P chemical shift (ppm) of pure phosphate standards (PA [N:P = 0], MAP [N:P = 1] and DAP [N:P = 2]) and their physical mixtures dissolved in D_2O solvent.

Table S1. Elemental analysis summary of phosphorus (P), hydrogen (H), nitrogen (N) and oxygen (O) content (wt%) and predicted N groups. Theoretical content (top value of most rows, regular font) is calculated from aggregate molecular formulae of pure and/or physically mixed reagent analytes. Experimental measurements (bottom value of most rows, ***bolded italic***) via ICP-OES (P) or CHN (H, N), or else estimated by difference (O). Predicted N groups indicate expected atomic N stoichiometry within analyte based on theoretical or experimental N content readouts.

Analyte	$P^{a,d}$ (wt%)	H^{b} (wt%)	N^{b} (wt%)	$O^{a,b,c}$ (wt%)	Predicted N groups[d]
H_3PO_4 [PA]	31.6	3.1	0.0 [d]	65.3	0.0 [d]
	34.1	***3.5***	***0.0***	***62.4***	***-0.04*** [e]
$(NH_4)H_2PO_4$ [MAP]	26.9	5.3	12.2 [d]	55.6	1.0 [d]
	28.2	***5.1***	***12.0***	***47.4***	***1.1***
$(NH_4)_2HPO_4$ [DAP]	23.4	6.9	21.2 [d]	48.5	2.0 [d]
	24.4	***6.9***	***21.3***	***47.4***	***2.0***
$H_4P_2O_7$ [PP]	34.8	2.3	0.0	62.9	0.0
	41.0	***2.8***	***-0.2***	***56.3***	***-0.06*** [e]
PA + MAP	29.2	4.2	6.4	60.2	0.5
(1:1 mol/mol)	***33.0***	***4.4***	***6.5***	***56.1***	***0.6***
PA + DAP	26.9	5.3	12.2	55.6	1.0
(1:1 mol/mol)	***29.8***	***5.3***	***12.4***	***52.6***	***1.1***
MAP + DAP	25.0	6.1	17.1	51.7	1.5
(1:1 mol/mol)	***38.2***	***5.5***	***16.6***	***39.8***	***1.5***
PA + MAP + DAP	27.1	5.2	11.8	55.9	1.0
(1:1:1 mol/mol/mol)	***27.9***	***5.3***	***12.3***	***54.6***	***1.1***
Sorption Expt. A1[f]	***27.0***	***6.1***	***15.3***	***51.6***	***1.4***
Sorption Expt. A2[f]	***24.4***	***6.0***	***14.6***	***54.9***	***1.3***
Sorption Expt. B[g]	***25.2***	***5.7***	***14.1***	***55.1***	***1.3***

[a] P content from ICP-OES. Despite baseline correction, experimental error may derive from non-zero P content in background signals.

[b] H, N content from CHN analysis. (All carbon (C) content is approximately zero by measurement.)

[c] O content inferred by difference from P and from H/N measurements.

[d] N groups calculated by interpolation of a linear fit (i.e., y_N [wt%] = m*[N:P] + b, m = 0.106, b = 0.0052, R^2 = 0.993) of theoretical N (wt%) and known N stoichiometry by chemical formula.

[e] Interpretted as zero.

[f] Replicate samples generated in two independent batch reactor trials. Experimental condition: 15.2 g DAP charged, 25–250°C ramp, 200 rpm stir rate. See **Section 2.1** in main text.

[g] Experimental condition: 7.44 g MAP and 8.56 g DAP charged (1:1 mol/mol), 25–250°C ramp, 200 rpm stir rate. See **Section 2.1** in main text.

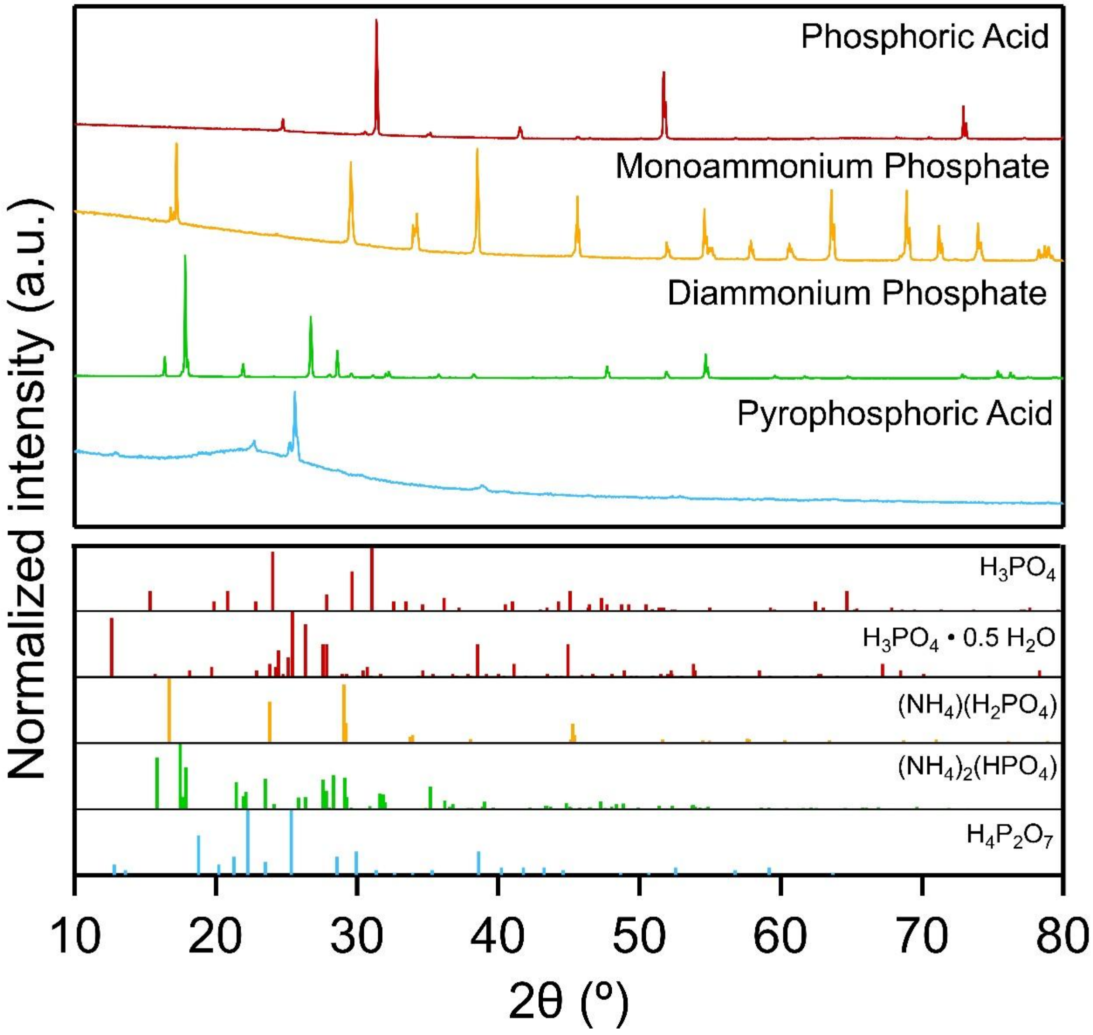

Figure S5. Powder X-ray diffraction patterns of phosphate standards, as received from vendor (top four panels). Diffraction patterns compared using PDF-2 2021 diffractogram database (bottom five panels).

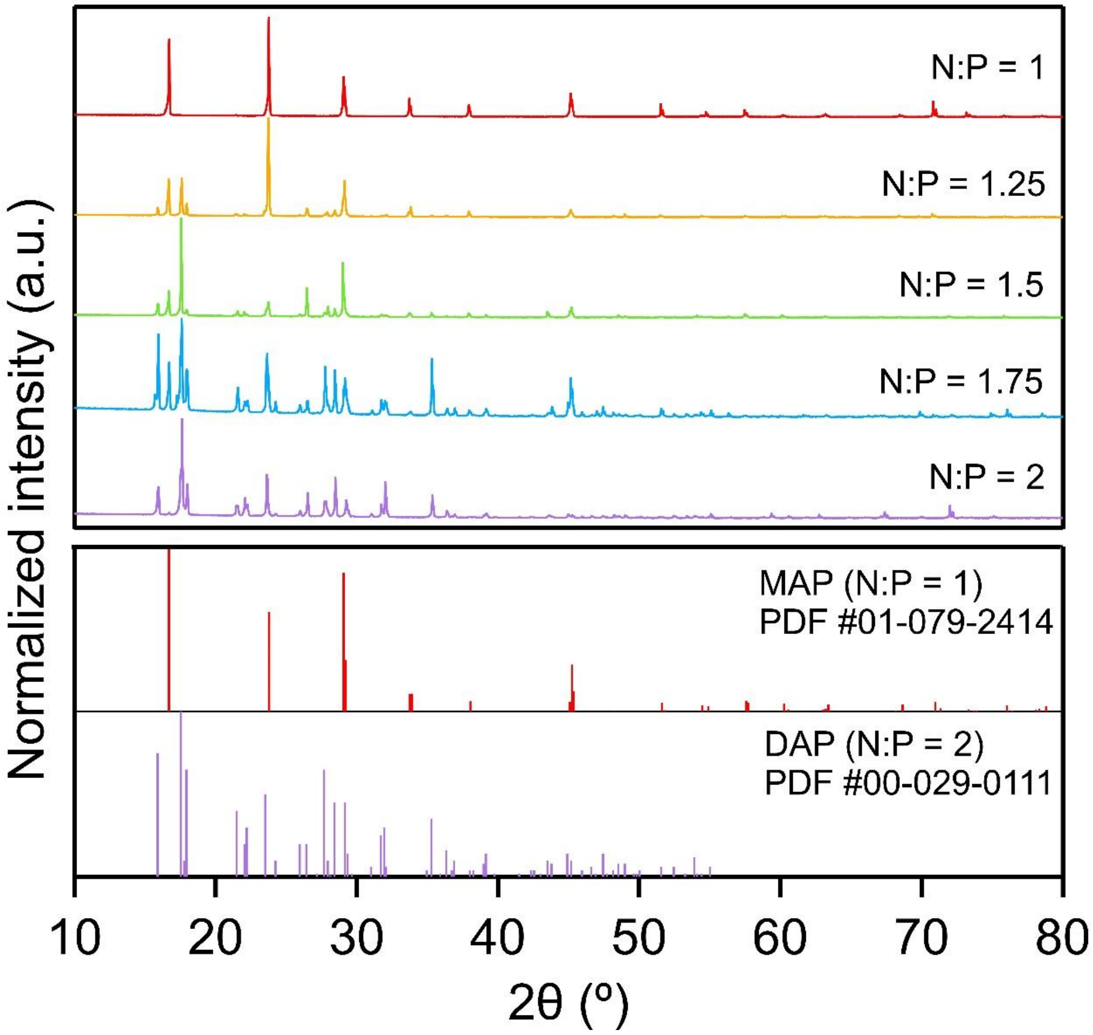

Figure S6. Powder X-ray diffraction patterns of MAP and DAP mixtures with targeted N:P ratios, ground in a mortar and pestle (top five panels). Diffractions patterns compared using PDF database diffractograms of MAP and DAP (bottom two panels).

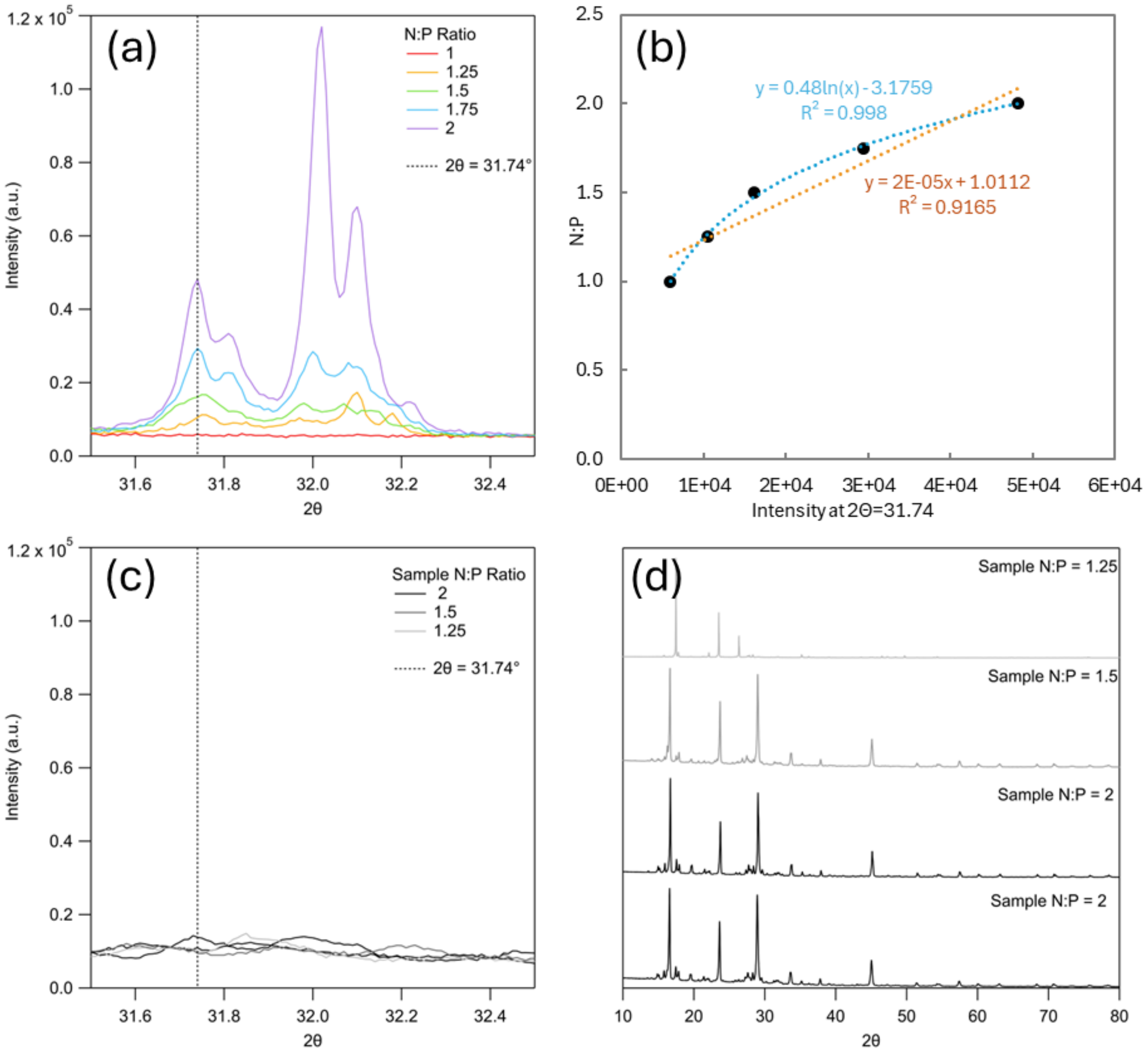

Figure S7. (a) Diffractogram raw signal for synthetic mixtures of MAP and DAP spanning N:P values between 1–2. Vertical dotted line indicates 2θ position of 31.74°. (b) Empirical logarithmic function and linear function fits of N:P as a function of pXRD peak intensity at 2θ = 31.74°. We note that application of the logarithmic fit to experimental samples yielded inconclusive results for unreliable N:P ratio prediction; see discussion in main text. (c) Diffractogram signal for four experimental samples of three different N:P ratios. No strong peak is visible at 2θ = 31.74°. (d) Full diffractogram pattern for the four samples of three different N:P ratios.

Process modeling and technoeconomic methods, assumptions and analysis

Development of conventional ammonia process flow diagram

The process flow diagram below in **Figure S8** represents the system boundary of the conventional ammonia production process modeled in Aspen Plus. The ammonia production rate is 100 tonnes d^{-1} (i.e., 36,500 tonnes yr^{-1}).

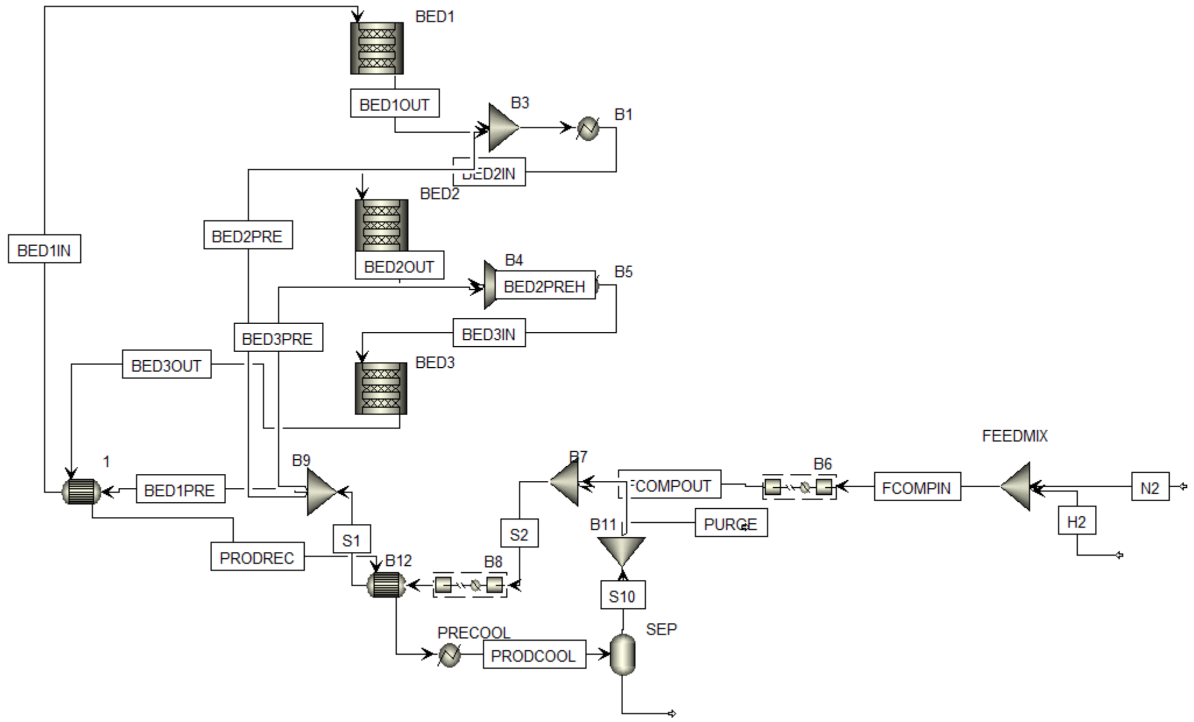

Figure S8. Conventional ammonia synthesis loop flow diagram via the Haber-Bosch process with H_2 and N_2 purchased at the plant gate.

H_2 and N_2 are fed to the process at a 3:1 stochiometric molar ratio. The production of N_2 and H_2 is not included in the system boundary to keep the ammonia production unit flexible and disconnected from upstream N_2 and H_2 production. However, we acknowledge that there are potential heat integration opportunities depending on the source of N_2 and H_2. By default, H_2 is assumed to be fed at 30 bar to represent PEM electrolysis and N_2 at 1 bar to represent PSA

pressures. The gases to a multi-stage centrifugal compressor and compressed to just under reactor pressure. A second recycle compressor brings the combined gas stream to the reactor pressure of 300 or 150 bar. Inter-coolers keep the temperature of each compression stage to below 150°C to prevent compressor oil breakdown and to increase compressor lifetime.[8]

The first point of heat integration in the process is to preheat the ammonia synthesis gas leaving block B8 with the unseparated reactor product gas via a counter-current shell and tube heat exchanger B12. The synthesis gas prefers to be higher temperature for reactor productivity and the product gas needs to be cooled for condensation. Following the feed preheater, the reactor inlet gas is split into 3 streams *BED1PRE*, *BED2PRE*, and *BED3PRE* where the number indicates which reactor bed the feed is fed to. This quench type converter design[9] is adopted in the M.W. Kellogg processes. By splitting the feed, fresh synthesis gas quenches moves through the 3 reactor beds improving conversion. Simultaneously, fresh synthesis gas shifts the equilibrium to the reactants, improving the driving force for the reaction. The exact feed split ratio selected in the model depends on the following rules of thumb listed below.

1. The quenched gas should not decimate the reaction rate within the reactor bed, which would occur if too much feed is sent to the 2nd or 3rd stage where the reactor temperature gain from the exothermic reactions isn't as significant, and
2. A larger portion of the feed should go through the first bed, which is farthest from equilibrium.

The feed fed to the first reactor bed is preheated to 370°C while the other feed streams are partially heated when mixed with the reactor bed effluents. The final reactor product exchanges heat with the feed gas and is cooled to 23°C to condense and separate ammonia from the recycle gases.

Development of sorption-based ammonia process

The process flow diagram in **Figure S9** represents the system boundary of the separation portion of the absorption enhanced ammonia synthesis process. The unit operations below are added prior to the condenser in the conventional ammonia process. We note that this is a preliminary design and is not optimized to reduce utility costs or capital costs. Given the emergent nature of this system, this section was designed with significant assumptions and caveats to mimic the experimental data where possible. The goal of this initial design was to identify potential issues with the sorption-based system that may not be easily identifiable without a full process simulation.

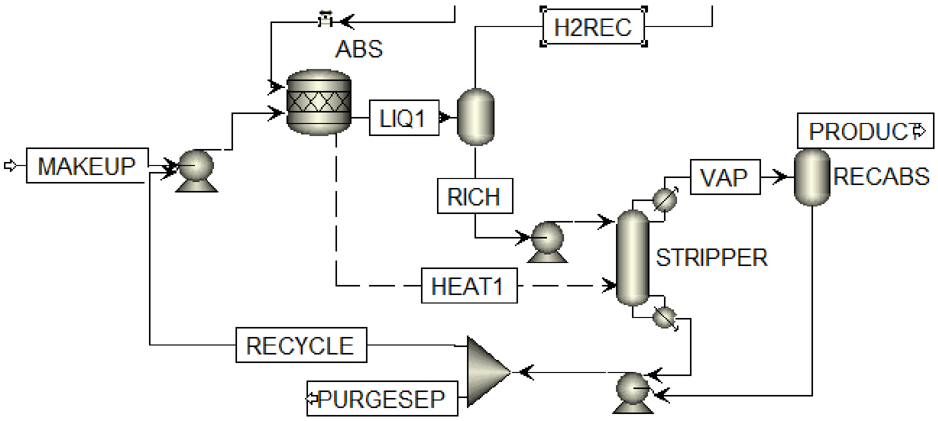

Figure S9. Liquid sorption-enhanced ammonia separation process segment flow diagram.

The hot products of the synthesis reactor are fed to the absorber operating isothermally at 200°C. The absorber equilibrium composition at these conditions is obtained from the experimental data. Assumptions used to develop preliminary models for absorption and desorption leverage the experimental data presented in **Section 3** of the main text. The NH_3-rich solvent is depressurized to the flash drum pressure of 10 bar to remove non-condensable components. The rich solvent is repressurized to the column pressure of 18.68 bar and fed to the top stage of the stripper. The ammonia product leaves the stripper as a vapor and the lean solvent is recycled back

to the absorber. A purge block is used to improve convergence. An optional flash block RECABS is specified to represent the condensation of the ammonia product vapor for ammonia liquefaction. The liquefaction of ammonia for storage and higher purities isn't modeled, as it is expected to be similar for both the conventional and absorption enhanced cases. Lastly, a makeup stream of phosphoric acid is used to address PA losses in the gaseous recycle and the purge stream.

Development of ammonia synthesis models

Aspen Plus specifications. The Redlich-Kwong-Soave-Boston-Mathias (RKS-BM) property method was selected to model ammonia synthesis. RKS-BM is selected to model the overall ammonia synthesis process as it is typically used for nonpolar or mildly polar mixtures of gases at higher temperatures and pressures. Parameters used in the RKS-BM are populated using databanks in Aspen Plus.

Reactor model. The ammonia reactor model consists of 3 adiabatic beds with intercoolers. The ammonia reactor model kinetics are described below in. The general form of LHHW kinetics in Aspen is described below. Ammonia synthesis kinetics from literature[10, 11] are adapted to meet this generic form.

$$k * e^{\frac{-E_A}{RT}} * \frac{k_f[\mathrm{A}]^a[\mathrm{B}]^b[\mathrm{C}]^c[\mathrm{D}]^d - k_b\,[\mathrm{A}]^a[\mathrm{B}]^b[\mathrm{C}]^c[\mathrm{D}]^d}{\Sigma\,\mathrm{Ki}\left(\Pi\, c_j^{v_j}\ \forall\ \mathrm{j}\in 1 \ldots \mathrm{n}\right)\forall\ \mathrm{i}\in 1 \ldots \mathrm{m}} \quad \text{(S1)}$$

$$\text{where } \ln(k_f) = A + B/T + C\ln(T) + DT \quad \text{(S2)}$$

The rate of ammonia formation can be described as

$$r_{NH3}\ (\mathrm{mol/m^3_{cat}s}) = k(p_{N2}K_a^2 - p^2_{NH3}/p^3_{H2}) / (1 + K_{NH3}p_{NH3}/p^{1.564}_{H2})^{1.28} \quad \text{(S3)}$$

$$\text{where } k = 1.096 * 10^{10}(mol/(m^3_{cat} * s * atm)) * e^{(-46737\ (J/Mol)/(R*T))} \quad (S4)$$

$$\log_{10} K_a = -2.69 * \log_{10}(T) - 5.52 * 10^{-5} T + 1.85 * 10^{-7} T^2 + (2001.6/T) + 2.6899 \quad (S5)$$

$$K_{NH3} = 2.94 * 10^{-4}\ atm^{(1-1.564)} * e^{(100628/(RT))} \quad (S6)$$

Eq. S3 consists of a kinetic factor k, driving force ($p_{N2}K_a^2 - p^2_{NH3}/p^3_{H2}$) and adsorption term ($(1 + K_{NH3}p_{NH3}/p^{1.564}_{H2})^{1.28}$.

The driving force expression consists of two terms k_f $[A]^a[B]^b[C]^c[D]^d$ and k_b $A]^a[B]^b[C]^c[D]^d$ which can be represented by **Eq. S3**. For Term 1, N_2 is raised to the power of 1 while k_f is equal to K_A^2. However, for Aspen Plus k_f must be in the form of **Eq. S2** for Aspen Plus kinetics. To address this a change of basis, we perform a regression from 50–600°C according to the following steps:

1. K_a^2 is calculated in 1°C intervals from 50–600°C using **Eq. S5**,
2. A regression for fitting K_a^2 vs (2) is done by minimizing the sum of residuals squared,
3. The resulting exponents are A = 21.46, B = 8942.65, C = -6.95, D = 0.0026, and
4. Post adjustment for units to Pa by adjusting A by a factor of E^(1/101325^2).

For Term 2, there is no driving force coefficient, and thus only powers are specified.

Reactor specifications. The adsorption term is described in the denominator of **Eq. S1**, where j is a component, v is the power, and i is a term in the expression. K is expressed in the form of **Eq. S2**. The denominator in equation **Eq. S3** and the equilibrium expression in **Eq. S6** are used to specify the adsorption term in Aspen Plus.

The reactor beds are sized by defining their space velocity. A space velocity is specified using Aspen Design Specs that iterates the reactor length to achieve a space velocity of 10000 h^{-1} for the entire 3-bed reactor (same residence time in each bed). For the synthesis loop, the reactor feed temperature is 370°C, the reactor pressure is 300 bar and the reactor volume is set to maintain

a space velocity of 10,000 hr^{-1} through each bed. The catalyst density is 3000 kg/m^3 with a shape factor of 1. The feed is split 0.45 to bed 1, 0.3 to bed 2 and 0.3 to bed 3. A summary results table of the bed conversion, NH_3 concentration in the outlet of each bed and the bed outlet temperature is presented below in **Table S2**.

Table S2. Adiabatic reactor stage specifications for the ammonia synthesis section.

Stage	Bed Outlet Temperature (°C)	Bed Conversion (%)	Effluent NH_3 Mole Fraction
Bed 1	455.7	10.4	0.114
Bed 2	447.9	10.0	0.146
Bed 3	441.9	9.6	0.168

Compressor model. Centrifugal compressors are specified using Aspen Plus built-in multistage compressor model. The compressors are assumed to be isentropic. An isentropic efficiency of 85% and mechanical efficiency of 95% is assumed for all compressors. The number of compression stages is selected to ensure that each individual stage does not exceed a compression ratio of 4. A pressure drop of 15 psi for each reactor bed and 10 psi across each heat exchanger is specified.

Heat exchanger model. Countercurrent heat exchangers are specified using an overall heat transfer coefficient and the log mean temperature difference (LMTD). The overall heat transfer coefficient for the gas-gas systems is specified to be 15 W/m^2K.[12] The minimum temperature approach is 10K. Cooling water is used to condense ammonia. The cooling water inlet temperature is 20°C, the cooling water outlet temperature is 25°C and the overall heat transfer coefficient is specified to be 150 W/m^2K.

Ammonia sorption assumptions. A simple model is used to provide a first order estimate of the feasibility of ammonia absorption and desorption using phosphoric acid given existing

experimental work completed. Continuing experimental work will produce experimental data that will lead to the development of future electrolyte based thermodynamic model (e.g., ENRTL) in Aspen Plus. Thus, several assumptions are necessary to estimate the system equilibrium using experimental data.

First, we assume that any mixture of phosphoric acid, ammonia, and ammonium phosphates will be at equilibrium. This assumption holds for the absorption of ammonia by phosphoric acid given the rapid nature of the acid–base reaction. There will be some error assuming equilibrium in the desorption of ammonia from ammonium phosphates given that temperature is the key driving force for desorption. To minimize this error, the stripping column is designed to operate around the maximum experimental operating temperature of 250°C. The desorption of ammonia is endothermic and thus by Le Chatelier's principle the desorption rate is likely higher by operating around the maximum operating temperature. The second major assumption is to assume that there is no MAP in the system as there is little available experimental data on the distribution of ammonium phosphates (see **Section 3.4**), and MAP properties are not available in Aspen Plus. Nonetheless, DAP is the desired equilibrium product (see **Sections 3.3–3.5** in main text) and thus is the targeted ammonium phosphate for the purposes of this analysis.

The third major assumption is to assume that absorption always occurs at around 175°C at which 1% of the total ammonia in the system will be desorbed as gaseous ammonia. This assumption is based on the experimental absorption data at 200°C for a N:P molar ratio of 2. The absorption process is assumed to occur isothermally at 200°C and is heat integrated with the reboiler on the desorption column. To simplify the analysis, the absorber is modeled with a "black-box" stochiometric reactor to represent the overall absorption of ammonia by phosphoric acid. In practice, the absorption process may consist of a stirred tank reactor and an absorption column in

series. The stirred tank reactor is used as a pre-neutralizer. The stirred tank reactor would be ideal for the initial neutralization effective given the high heat generation, mixing and process controls associated with the neutralization reaction. The following column would be used to maximize the absorption of ammonia. The first-order cost estimate in the economic modeling section assumes a stirred tank reactor and column for the absorber. The fourth and final major assumption is that DAP does not exhibit a vapor pressure.

Ammonia sorption modeling. Solubility estimates for ammonia in a mixture of phosphoric acid and ammonium phosphates are described using Henry's law. The Henry's constant can be expressed as a function of absolute temperature (K) in Aspen Plus using **Eq. S7** below:

$$\ln(H(T)) = A + B/T + C*\mathrm{Ln}(T) + D*T \qquad \text{(S7)}$$

Dimensionless Henry's law constants were obtained from ReaxFF MD (see **Section 3.6**, **Table 2**). Values between 180–240°C were extracted for the following fitting exercise and are assumed to represent mixtures of phosphoric acid and ammonium phosphates at an N:P ratio of 2. The dimensionless Henry's constant is converted to a pressure basis by assuming that ammonia behaves as an ideal gas around absorption and stripping temperatures and pressures. This assumption can be made as operating conditions of 250C and <20 bar are sufficiently far from the critical point of ammonia. The results of a regression of experimental Henry's constants is presented below in **Table S3**.

Table S3. Regressed values of **Eq. S7** to fit a temperature-dependent function of Henry's law constants.

Parameter	Regressed Value
A	-13.89
B	899.99
C	0.35
D	0.010

The absorber is modeled using a RSTOIC block. The fractional conversion of the absorption reaction is varied until 7% of the ammonia-related compounds in the product stream exist as ammonia using a design specification. This step represents the absorption of ammonia at 200°C and 145 bar. The heat generated in this reaction is supplied to the reboiler.

An adiabatic flash drum is used to separate uncondensed gases comprising of H_2, N_2, NH_3 and other trace gases at 10 bar. Ideally, desorption be performed under the same pressure as the synthesis loop and the absorber. This would reduce the work required to recompress the recycle gases back to synthesis pressures. However, in the current model trace but non-negligible amounts of phosphoric acid ($y_{H3PO4} = 0.005$) are present in the recycle gas. Further experimental studies would be needed to evaluate whether non-negligible amounts of gaseous phosphoric acid exist when absorption and desorption occurs at synthesis pressures.

The stripper column is specified as a 10-equilibrium-stage column operating at a pressure of 18.68 bar. No pressure drop is assumed through the column. The distillate rate is 0.7 kmol/sec and the reboiler duty is supplied by the absorber. The equilibrium for the decomposition of DAP into ammonia and phosphoric acid is estimated by regressing the experimental equilibrium data at N:P ratios of 2 between 175°C to 250°C into the form of **Eq. S8** below; the regressed parameters are presented in **Table S4**. Finally, complete material and energy balances for the conventional and sorption-enhanced process models are reported in **Table S5** and **Table S6**, respectively.

$$\ln(K_{eq}) = A + B/T + C * \ln(T) \qquad (S8)$$

Table S4. Regressed values of **Eq. S8** to fit a temperature-dependent function of the DAP decomposition equilibrium. A mean absolute error of 1.9% was observed following the regression.

Parameter	Regressed Value
A	-50.97
B	-70.87
C	12.78

Table S5. Material and energy balances for the 150-bar conventional ammonia production model

Stream Name	Units	BED1IN	BED1OUT	BED1PRE	BED1PREH	BED2IN	BED2OUT	BED2PRE	BED2PREH	BED3IN	BED3OUT	BED3PRE
**From**		1	BED1	B9	B3	B1	BED2	B9	B4	B5	BED3	B9
**To**		BED1	B3	1	B1	BED2	B4	B3	B5	BED3	1	B4
**Temperature**	K	6.43E+02	7.37E+02	5.23E+02	6.60E+02	6.43E+02	7.22E+02	5.23E+02	6.62E+02	6.43E+02	7.12E+02	5.23E+02
**Pressure**	N/sqm	1.50E+07	1.50E+07	1.50E+07	1.50E+07	1.50E+07	1.50E+07	1.50E+07	1.50E+07	1.50E+07	1.50E+07	1.50E+07
**Molar Enthalpy**	J/kmol	7.40E+06	7.82E+06	3.67E+06	6.29E+06	5.75E+06	6.04E+06	3.67E+06	5.29E+06	4.69E+06	4.89E+06	3.67E+06
**Mass Enthalpy**	J/kg	8.12E+05	8.12E+05	4.03E+05	6.66E+05	6.10E+05	6.10E+05	4.03E+05	5.48E+05	4.86E+05	4.86E+05	4.03E+05
**Molar Entropy**	J/kmol-K	-1.91E+04	-1.94E+04	-2.55E+04	-2.12E+04	-2.20E+04	-2.26E+04	-2.55E+04	-2.31E+04	-2.40E+04	-2.47E+04	-2.55E+04
**Mass Entropy**	J/kg-K	-2.09E+03	-2.02E+03	-2.80E+03	-2.25E+03	-2.33E+03	-2.28E+03	-2.80E+03	-2.39E+03	-2.49E+03	-2.45E+03	-2.80E+03
**Molar Density**	kmol/cum	2.66E+00	2.34E+00	3.24E+00	2.59E+00	2.66E+00	2.38E+00	3.24E+00	2.59E+00	2.66E+00	2.41E+00	3.24E+00
**Mass Density**	kg/cum	2.42E+01	2.25E+01	2.95E+01	2.45E+01	2.51E+01	2.36E+01	2.95E+01	2.50E+01	2.57E+01	2.43E+01	2.95E+01
**Mole Flows**	kmol/sec	3.23E+00	3.05E+00	3.23E+00	4.85E+00	4.85E+00	4.62E+00	1.79E+00	6.77E+00	6.77E+00	6.49E+00	2.15E+00
**NH_3**	kmol/sec	2.28E-01	4.03E-01	2.28E-01	5.30E-01	5.30E-01	7.57E-01	1.27E-01	9.09E-01	9.09E-01	1.19E+00	1.52E-01
**H_2**	kmol/sec	2.25E+00	1.99E+00	2.25E+00	3.24E+00	3.24E+00	2.90E+00	1.25E+00	4.40E+00	4.40E+00	3.97E+00	1.50E+00
**N_2**	kmol/sec	7.49E-01	6.62E-01	7.49E-01	1.08E+00	1.08E+00	9.64E-01	4.16E-01	1.46E+00	1.46E+00	1.32E+00	4.99E-01
**Mole Fractions**												
**NH_3**		7.06E-02	1.32E-01	7.06E-02	1.09E-01	1.09E-01	1.64E-01	7.06E-02	1.34E-01	1.34E-01	1.84E-01	7.06E-02
**H_2**		6.97E-01	6.51E-01	6.97E-01	6.68E-01	6.68E-01	6.27E-01	6.97E-01	6.50E-01	6.50E-01	6.12E-01	6.97E-01
**N_2**		2.32E-01	2.17E-01	2.32E-01	2.22E-01	2.22E-01	2.09E-01	2.32E-01	2.16E-01	2.16E-01	2.04E-01	2.32E-01
**Mass Flows**	kg/sec	2.94E+01	2.94E+01	2.94E+01	4.57E+01	4.57E+01	4.57E+01	1.63E+01	6.54E+01	6.54E+01	6.54E+01	1.96E+01
**NH_3**	kg/sec	3.88E+00	6.87E+00	3.88E+00	9.02E+00	9.02E+00	1.29E+01	2.16E+00	1.55E+01	1.55E+01	2.03E+01	2.59E+00
**H_2**	kg/sec	4.54E+00	4.01E+00	4.54E+00	6.53E+00	6.53E+00	5.84E+00	2.52E+00	8.87E+00	8.87E+00	8.01E+00	3.03E+00
**N_2**	kg/sec	2.10E+01	1.85E+01	2.10E+01	3.02E+01	3.02E+01	2.70E+01	1.17E+01	4.10E+01	4.10E+01	3.70E+01	1.40E+01
**Mass Fractions**												
**NH_3**		1.32E-01	2.33E-01	1.32E-01	1.97E-01	1.97E-01	2.82E-01	1.32E-01	2.37E-01	2.37E-01	3.11E-01	1.32E-01
**H_2**		1.54E-01	1.36E-01	1.54E-01	1.43E-01	1.43E-01	1.28E-01	1.54E-01	1.36E-01	1.36E-01	1.23E-01	1.54E-01
**N_2**		7.14E-01	6.30E-01	7.14E-01	6.60E-01	6.60E-01	5.90E-01	7.14E-01	6.27E-01	6.27E-01	5.67E-01	7.14E-01
**Volume Flow**	cum/sec	1.22E+00	1.31E+00	9.97E-01	1.87E+00	1.82E+00	1.94E+00	5.54E-01	2.62E+00	2.55E+00	2.69E+00	6.65E-01

Table S5. (cont'd)

Stream Name	Units	FCOMPIN	FCOMPOUT	FEEDTOR	H2	N2	PROD	PRODCOOL	PRODREC	PURGE	S1	S2	S3	S9	S10
**From**		FEEDMIX	B6	B8			SEP	PRECOOL	1	B11	B12	B7	B12	B11	SEP
**To**		B6	B7	B12	FEEDMIX	FEEDMIX		SEP	B12		B9	B8	PRECOOL	B7	B11
**Temperature**	K	2.94E+02	4.23E+02	4.23E+02	2.93E+02	2.93E+02	2.93E+02	2.93E+02	6.57E+02	2.93E+02	5.23E+02	3.18E+02	5.54E+02	2.93E+02	2.93E+02
**Pressure**	N/sqm	1.01E+05	1.40E+07	1.50E+07	3.00E+06	1.01E+05	1.50E+07	1.50E+07	1.50E+07	1.50E+07	1.50E+07	1.40E+07	1.50E+07	1.50E+07	1.50E+07
**Molar Enthalpy**	J/kmol	-1.27E+05	3.81E+06	5.69E+05	-1.19E+05	-1.52E+05	-6.67E+07	-1.10E+07	3.04E+06	-4.42E+06	3.67E+06	-2.71E+06	-3.92E+05	-4.42E+06	-4.42E+06
**Mass Enthalpy**	J/kg	-1.49E+04	4.47E+05	6.25E+04	-5.88E+04	-5.43E+03	-3.94E+06	-1.09E+06	3.02E+05	-4.77E+05	4.03E+05	-2.98E+05	-3.89E+04	-4.77E+05	-4.77E+05
**Molar Entropy**	J/kmol-K	4.24E+03	-2.63E+04	-3.21E+04	-2.87E+04	-5.15E+02	-1.89E+05	-6.04E+04	-2.74E+04	-4.53E+04	-2.55E+04	-4.03E+04	-3.30E+04	-4.53E+04	-4.53E+04
**Mass Entropy**	J/kg-K	4.98E+02	-3.09E+03	-3.52E+03	-1.43E+04	-1.84E+01	-1.12E+04	-6.00E+03	-2.72E+03	-4.89E+03	-2.80E+03	-4.43E+03	-3.28E+03	-4.89E+03	-4.89E+03
**Molar Density**	kmol/cum	4.15E-02	3.70E+00	3.97E+00	1.21E+00	4.16E-02	2.87E+01	6.27E+00	2.61E+00	5.74E+00	3.24E+00	4.93E+00	3.08E+00	5.74E+00	5.74E+00
**Mass Density**	kg/cum	3.53E-01	3.15E+01	3.62E+01	2.43E+00	1.16E+00	4.86E+02	6.31E+01	2.63E+01	5.32E+01	2.95E+01	4.49E+01	3.10E+01	5.32E+01	5.32E+01
**Mole Flows**	kmol/sec	1.49E+00	1.49E+00	7.18E+00	1.11E+00	3.72E-01	6.83E-01	6.49E+00	6.49E+00	1.16E-01	7.18E+00	7.18E+00	6.49E+00	5.69E+00	5.81E+00
**NH_3**	kmol/sec	0.00E+00	0.00E+00	5.06E-01	0.00E+00	0.00E+00	6.76E-01	1.19E+00	1.19E+00	1.03E-02	5.06E-01	5.06E-01	1.19E+00	5.06E-01	5.17E-01
**H_2**	kmol/sec	1.11E+00	1.11E+00	5.00E+00	1.11E+00	0.00E+00	5.28E-03	3.97E+00	3.97E+00	7.94E-02	5.00E+00	5.00E+00	3.97E+00	3.89E+00	3.97E+00
**N_2**	kmol/sec	3.72E-01	3.72E-01	1.66E+00	0.00E+00	3.72E-01	1.80E-03	1.32E+00	1.32E+00	2.64E-02	1.66E+00	1.66E+00	1.32E+00	1.29E+00	1.32E+00
**Mole Fractions**															
**NH_3**		0.00E+00	0.00E+00	7.06E-02	0.00E+00	0.00E+00	9.90E-01	1.84E-01	1.84E-01	8.90E-02	7.06E-02	7.06E-02	1.84E-01	8.90E-02	8.90E-02
**H_2**		7.50E-01	7.50E-01	6.97E-01	1.00E+00	0.00E+00	7.72E-03	6.12E-01	6.12E-01	6.84E-01	6.97E-01	6.97E-01	6.12E-01	6.84E-01	6.84E-01
**N_2**		2.50E-01	2.50E-01	2.32E-01	0.00E+00	1.00E+00	2.64E-03	2.04E-01	2.04E-01	2.27E-01	2.32E-01	2.32E-01	2.04E-01	2.27E-01	2.27E-01
**Mass Flows**	kg/sec	1.27E+01	1.27E+01	6.54E+01	2.25E+00	1.04E+01	1.16E+01	6.54E+01	6.54E+01	1.08E+00	6.54E+01	6.54E+01	6.54E+01	5.27E+01	5.38E+01
**NH_3**	kg/sec	0.00E+00	0.00E+00	8.62E+00	0.00E+00	0.00E+00	1.15E+01	2.03E+01	2.03E+01	1.76E-01	8.62E+00	8.62E+00	2.03E+01	8.62E+00	8.80E+00
**H_2**	kg/sec	2.25E+00	2.25E+00	1.01E+01	2.25E+00	0.00E+00	1.06E-02	8.01E+00	8.01E+00	1.60E-01	1.01E+01	1.01E+01	8.01E+00	7.84E+00	8.00E+00
**N_2**	kg/sec	1.04E+01	1.04E+01	4.66E+01	0.00E+00	1.04E+01	5.05E-02	3.70E+01	3.70E+01	7.39E-01	4.66E+01	4.66E+01	3.70E+01	3.62E+01	3.70E+01
**Mass Fractions**															
**NH_3**		0.00E+00	0.00E+00	1.32E-01	0.00E+00	0.00E+00	9.95E-01	3.11E-01	3.11E-01	1.64E-01	1.32E-01	1.32E-01	3.11E-01	1.64E-01	1.64E-01
**H_2**		1.78E-01	1.78E-01	1.54E-01	1.00E+00	0.00E+00	9.18E-04	1.23E-01	1.23E-01	1.49E-01	1.54E-01	1.54E-01	1.23E-01	1.49E-01	1.49E-01
**N_2**		8.22E-01	8.22E-01	7.14E-01	0.00E+00	1.00E+00	4.36E-03	5.67E-01	5.67E-01	6.88E-01	7.14E-01	7.14E-01	5.67E-01	6.88E-01	6.88E-01
**Volume Flow**	cum/sec	3.58E+01	4.01E-01	1.81E+00	9.23E-01	8.94E+00	2.38E-02	1.04E+00	2.49E+00	2.02E-02	2.22E+00	1.46E+00	2.11E+00	9.91E-01	1.01E+00

Table S6. Material and energy balances for the 150-bar sorption-enhanced ammonia production model

Stream Name	Units	BED1IN	BED1OUT	BED1PRE	BED1PREH	BED2IN	BED2OUT	BED2PRE	BED2PREH	BED3IN	BED3OUT	BED3PRE	DAPREC	FCOMPIN	FCOMPOUT	FEEDTOR	H2
**From**		1	BED1	B9	B3	B1	BED2	B9	B4	B5	BED3	B9	B13	FEEDMIX	B6	B8	
**To**		BED1	B3	1	B1	BED2	B4	B3	B5	BED3	1	B4	ABS	B6	B7	B12	FEEDMIX
**Temperature**	K	6.43E+02	7.74E+02	5.23E+02	6.83E+02	6.43E+02	7.54E+02	5.23E+02	6.83E+02	6.43E+02	7.41E+02	5.23E+02	7.41E+02	2.94E+02	4.23E+02	4.23E+02	2.93E+02
**Pressure**	N/sqm	1.50E+07	1.51E+07	1.50E+07	1.50E+07	1.49E+07	1.50E+07	1.50E+07	1.50E+07	1.49E+07	1.50E+07	1.50E+07	1.46E+07	1.01E+05	1.40E+07	1.50E+07	3.00E+06
**Molar Enthalpy**	J/kmol	9.58E+06	1.03E+07	5.99E+06	8.70E+06	7.46E+06	7.97E+06	5.99E+06	7.33E+06	6.05E+06	6.42E+06	5.99E+06	-1.13E+09	-1.27E+05	3.81E+06	2.97E+06	-1.19E+05
**Mass Enthalpy**	J/kg	1.15E+06	1.15E+06	7.16E+05	9.92E+05	8.51E+05	8.51E+05	7.16E+05	8.10E+05	6.69E+05	6.69E+05	7.16E+05	-1.16E+07	-1.49E+04	4.47E+05	3.55E+05	-5.88E+04
**Molar Entropy**	J/kmol-K	-1.47E+04	-1.43E+04	-2.09E+04	-1.61E+04	-1.79E+04	-1.83E+04	-2.09E+04	-1.84E+04	-2.03E+04	-2.10E+04	-2.09E+04	-3.48E+05	4.24E+03	-2.63E+04	-2.73E+04	-2.87E+04
**Mass Entropy**	J/kg-K	-1.76E+03	-1.59E+03	-2.50E+03	-1.83E+03	-2.04E+03	-1.95E+03	-2.50E+03	-2.04E+03	-2.25E+03	-2.18E+03	-2.50E+03	-3.59E+03	4.98E+02	-3.09E+03	-3.27E+03	-1.43E+04
**Molar Density**	kmol/cum	2.66E+00	2.24E+00	3.23E+00	2.51E+00	2.65E+00	2.29E+00	3.23E+00	2.51E+00	2.65E+00	2.33E+00	3.23E+00	5.00E+00	4.15E-02	3.70E+00	3.95E+00	1.21E+00
**Mass Density**	kg/cum	2.22E+01	2.02E+01	2.70E+01	2.20E+01	2.32E+01	2.14E+01	2.70E+01	2.27E+01	2.39E+01	2.23E+01	2.70E+01	4.84E+02	3.53E-01	3.15E+01	3.30E+01	2.43E+00
**Mole Flows**	kmol/sec	2.35E+00	2.18E+00	2.35E+00	3.48E+00	3.48E+00	3.26E+00	1.31E+00	4.83E+00	4.83E+00	4.55E+00	1.57E+00	1.96E+01	1.49E+00	1.49E+00	5.22E+00	1.11E+00
**NH_3**	kmol/sec	8.36E-03	1.79E-01	8.36E-03	1.84E-01	1.84E-01	4.07E-01	4.64E-03	4.13E-01	4.13E-01	6.90E-01	5.57E-03	5.94E-01	0.00E+00	0.00E+00	1.86E-02	0.00E+00
**H_2**	kmol/sec	1.78E+00	1.52E+00	1.78E+00	2.51E+00	2.51E+00	2.17E+00	9.87E-01	3.36E+00	3.36E+00	2.94E+00	1.18E+00	1.46E-13	1.11E+00	1.11E+00	3.95E+00	1.11E+00
**N_2**	kmol/sec	5.64E-01	4.78E-01	5.64E-01	7.91E-01	7.91E-01	6.80E-01	3.13E-01	1.06E+00	1.06E+00	9.17E-01	3.76E-01	6.65E-14	3.72E-01	3.72E-01	1.25E+00	0.00E+00
**H_3PO_4**	kmol/sec	1.34E-03	1.34E-03	1.34E-03	2.08E-03	2.08E-03	2.08E-03	7.43E-04	2.97E-03	2.97E-03	2.97E-03	8.92E-04	1.83E+01	0.00E+00	0.00E+00	2.97E-03	0.00E+00
**$(NH_4)_2HPO_4$**	kmol/sec	3.94E-08	3.94E-08	3.94E-08	6.13E-08	6.13E-08	6.13E-08	2.19E-08	8.76E-08	8.76E-08	8.76E-08	2.63E-08	6.88E-01	0.00E+00	0.00E+00	8.76E-08	0.00E+00
**Mole Fractions**																	
**NH_3**		3.56E-03	8.23E-02	3.56E-03	5.28E-02	5.28E-02	1.25E-01	3.56E-03	8.55E-02	8.55E-02	1.52E-01	3.56E-03	3.03E-02	0.00E+00	0.00E+00	3.56E-03	0.00E+00
**H_2**		7.56E-01	6.98E-01	7.56E-01	7.19E-01	7.19E-01	6.66E-01	7.56E-01	6.95E-01	6.95E-01	6.46E-01	7.56E-01	7.42E-15	7.50E-01	7.50E-01	7.56E-01	1.00E+00
**N_2**		2.40E-01	2.19E-01	2.40E-01	2.27E-01	2.27E-01	2.08E-01	2.40E-01	2.19E-01	2.19E-01	2.02E-01	2.40E-01	3.39E-15	2.50E-01	2.50E-01	2.40E-01	0.00E+00
**H_3PO_4**		5.69E-04	6.14E-04	5.69E-04	5.97E-04	5.97E-04	6.38E-04	5.69E-04	6.16E-04	6.16E-04	6.53E-04	5.69E-04	9.35E-01	0.00E+00	0.00E+00	5.69E-04	0.00E+00
**$(NH_4)_2HPO_4$**		1.68E-08	1.81E-08	1.68E-08	1.76E-08	1.76E-08	1.88E-08	1.68E-08	1.81E-08	1.81E-08	1.92E-08	1.68E-08	3.51E-02	0.00E+00	0.00E+00	1.68E-08	0.00E+00
**Mass Flows**	kg/sec	1.96E+01	1.96E+01	1.96E+01	3.06E+01	3.06E+01	3.06E+01	1.09E+01	4.37E+01	4.37E+01	4.37E+01	1.31E+01	1.90E+03	1.27E+01	1.27E+01	4.37E+01	2.25E+00
**NH_3**	kg/sec	1.42E-01	3.05E+00	1.42E-01	3.13E+00	3.13E+00	6.93E+00	7.91E-02	7.03E+00	7.03E+00	1.17E+01	9.49E-02	1.01E+01	0.00E+00	0.00E+00	3.16E-01	0.00E+00
**H_2**	kg/sec	3.58E+00	3.06E+00	3.58E+00	5.05E+00	5.05E+00	4.38E+00	1.99E+00	6.77E+00	6.77E+00	5.93E+00	2.39E+00	2.93E-13	2.25E+00	2.25E+00	7.96E+00	2.25E+00
**N_2**	kg/sec	1.58E+01	1.34E+01	1.58E+01	2.22E+01	2.22E+01	1.90E+01	8.77E+00	2.96E+01	2.96E+01	2.57E+01	1.05E+01	1.86E-12	1.04E+01	1.04E+01	3.51E+01	0.00E+00
**H_3PO_4**	kg/sec	1.31E-01	1.31E-01	1.31E-01	2.04E-01	2.04E-01	2.04E-01	7.28E-02	2.91E-01	2.91E-01	2.91E-01	8.74E-02	1.80E+03	0.00E+00	0.00E+00	2.91E-01	0.00E+00
**$(NH_4)_2HPO_4$**	kg/sec	5.20E-06	5.20E-06	5.20E-06	8.10E-06	8.10E-06	8.10E-06	2.89E-06	1.16E-05	1.16E-05	1.16E-05	3.47E-06	9.08E+01	0.00E+00	0.00E+00	1.16E-05	0.00E+00

**Mass Fractions**																	
**NH_3**		7.25E-03	1.55E-01	7.25E-03	1.02E-01	1.02E-01	2.27E-01	7.25E-03	1.61E-01	1.61E-01	2.69E-01	7.25E-03	5.33E-03	0.00E+00	0.00E+00	7.25E-03	0.00E+00
**H_2**		1.82E-01	1.56E-01	1.82E-01	1.65E-01	1.65E-01	1.43E-01	1.82E-01	1.55E-01	1.55E-01	1.36E-01	1.82E-01	1.55E-16	1.78E-01	1.78E-01	1.82E-01	1.00E+00
**N_2**		8.04E-01	6.82E-01	8.04E-01	7.25E-01	7.25E-01	6.23E-01	8.04E-01	6.77E-01	6.77E-01	5.88E-01	8.04E-01	9.81E-16	8.22E-01	8.22E-01	8.04E-01	0.00E+00
**H_3PO_4**		6.67E-03	6.67E-03	6.67E-03	6.67E-03	6.67E-03	6.67E-03	6.67E-03	6.67E-03	6.67E-03	6.67E-03	6.67E-03	9.47E-01	0.00E+00	0.00E+00	6.67E-03	0.00E+00
**$(NH_4)_2HPO_4$**		2.65E-07	2.65E-07	2.65E-07	2.65E-07	2.65E-07	2.65E-07	2.65E-07	2.65E-07	2.65E-07	2.65E-07	2.65E-07	4.79E-02	0.00E+00	0.00E+00	2.65E-07	0.00E+00
**Volume Flow**	cum/sec	8.85E-01	9.72E-01	7.27E-01	1.39E+00	1.32E+00	1.43E+00	4.04E-01	1.93E+00	1.82E+00	1.95E+00	4.85E-01	3.92E+00	3.58E+01	4.01E-01	1.32E+00	9.23E-01

Table S6. (cont'd)

LIQ1	LIQ2	LIQ3	LIQ4	MAKEUP	N2	PROD	PRODREC	PURGESEP	RECYCLE	RPROD	S1	S2	S4	S5	S8	S9	S12	S21	S29	VAP
**Stream Name**	Units	STRIPPER	B20			RECABS	1	B16	B16	B14	B12	B7	B10	B12	B8	B11	ABSFLASH	RECABS	B23	STRIPPER
**From**		B20	B16	B13	FEEDMIX		B12		B13	ABS	B9	B8	STRIPPER	B14		B7	B23	B20	B11	RECABS
**To**		7.27E+02	7.35E+02	2.98E+02	2.93E+02	4.44E+02	6.85E+02	7.35E+02	7.35E+02	5.80E+02	5.23E+02	4.51E+02	4.60E+02	5.79E+02	4.23E+02	4.60E+02	4.60E+02	4.44E+02	4.60E+02	4.44E+02
**Temperature**	K	1.87E+06	1.45E+07	1.01E+05	1.01E+05	1.87E+06	1.50E+07	1.45E+07	1.45E+07	1.45E+07	1.50E+07	1.00E+06	1.87E+06	1.49E+07	3.87E+06	1.00E+06	1.00E+06	1.87E+06	1.00E+06	1.87E+06
**Pressure**	N/sqm	-1.13E+09	-1.13E+09	-1.25E+09	-1.52E+05	-3.92E+07	4.57E+06	-1.13E+09	-1.13E+09	1.11E+06	5.99E+06	-3.83E+05	-1.22E+09	1.11E+06	-1.21E+09	-2.04E+06	-2.04E+06	-1.05E+09	-2.04E+06	-3.92E+07
**Molar Enthalpy**	J/kmol	-1.17E+07	-1.17E+07	-1.27E+07	-5.43E+03	-2.39E+06	4.76E+05	-1.17E+07	-1.17E+07	1.15E+05	7.16E+05	-4.42E+04	-1.22E+07	1.15E+05	-1.25E+07	-2.34E+05	-2.34E+05	-1.20E+07	-2.34E+05	-2.39E+06
**Mass Enthalpy**	J/kg	-3.52E+05	-3.52E+05	-3.89E+05	-5.15E+02	-9.68E+04	-2.35E+04	-3.52E+05	-3.52E+05	-2.87E+04	-2.09E+04	-3.40E+03	-5.73E+05	-2.90E+04	-3.41E+05	-3.29E+03	-3.29E+03	-5.10E+05	-3.29E+03	-9.68E+04
**Molar Entropy**	J/kmol-K	-3.64E+03	-3.64E+03	-3.97E+03	-1.84E+01	-5.89E+03	-2.45E+03	-3.64E+03	-3.64E+03	-2.99E+03	-2.50E+03	-3.93E+02	-5.73E+03	-3.02E+03	-3.52E+03	-3.77E+02	-3.77E+02	-5.79E+03	-3.77E+02	-5.89E+03
**Mass Entropy**	J/kg-K	4.78E+00	5.02E+00	6.27E+00	4.16E-02	5.23E-01	2.50E+00	5.02E+00	5.02E+00	2.85E+00	3.23E+00	2.65E-01	5.59E+00	2.93E+00	6.02E+00	2.60E-01	2.60E-01	6.28E+00	2.60E-01	5.23E-01
**Molar Density**	kmol/cum	4.62E+02	4.86E+02	6.15E+02	1.16E+00	8.60E+00	2.40E+01	4.86E+02	4.86E+02	2.74E+01	2.70E+01	2.30E+00	5.59E+02	2.81E+01	5.83E+02	2.27E+00	2.27E+00	5.53E+02	2.27E+00	8.60E+00
**Mass Density**	kg/cum	1.96E+01	1.96E+01	4.00E-01	3.72E-01	7.00E-01	4.55E+00	3.92E-01	1.92E+01	4.55E+00	5.22E+00	5.24E+00	1.91E+01	4.55E+00	1.81E-02	3.75E+00	3.83E+00	3.04E-10	3.83E+00	7.00E-01
**Mole Flows**	kmol/sec	6.06E-01	6.06E-01	0.00E+00	0.00E+00	6.30E-01	6.90E-01	1.21E-02	5.94E-01	6.90E-01	1.86E-02	1.86E-02	7.42E-03	6.90E-01	2.72E-05	1.86E-02	1.90E-02	4.44E-11	1.90E-02	6.30E-01
**NH_3**	kmol/sec	6.30E-15	1.49E-13	0.00E+00	0.00E+00	5.00E-02	2.94E+00	2.97E-15	1.46E-13	2.94E+00	3.95E+00	3.95E+00	5.00E-02	2.94E+00	1.55E-04	2.83E+00	2.89E+00	1.42E-13	2.89E+00	5.00E-02
**H_2**	kmol/sec	7.73E-15	6.78E-14	0.00E+00	3.72E-01	1.79E-02	9.17E-01	1.36E-15	6.65E-14	9.17E-01	1.25E+00	1.25E+00	1.79E-02	9.17E-01	6.15E-05	8.81E-01	8.99E-01	6.01E-14	8.99E-01	1.79E-02
**N_2**	kmol/sec	1.83E+01	1.83E+01	4.00E-01	0.00E+00	1.68E-03	2.97E-03	3.66E-01	1.79E+01	2.97E-03	2.97E-03	2.08E-02	1.77E+01	2.97E-03	1.79E-02	2.08E-02	2.12E-02	2.41E-10	2.12E-02	1.68E-03
**H_3PO_4**	kmol/sec	7.02E-01	7.02E-01	0.00E+00	0.00E+00	2.83E-06	8.76E-08	1.40E-02	6.88E-01	8.76E-08	8.76E-08	3.70E-05	1.32E+00	8.76E-08	3.69E-05	3.70E-05	3.78E-05	1.74E-11	3.78E-05	2.83E-06
**$(NH_4)_2HPO_4$**	kmol/sec																			
**Mole Fractions**		3.09E-02	3.09E-02	0.00E+00	0.00E+00	9.01E-01	1.52E-01	3.09E-02	3.09E-02	1.52E-01	3.56E-03	3.55E-03	3.89E-04	1.52E-01	1.50E-03	4.96E-03	4.96E-03	1.46E-01	4.96E-03	9.01E-01
**NH_3**		3.22E-16	7.58E-15	0.00E+00	0.00E+00	7.14E-02	6.46E-01	7.58E-15	7.58E-15	6.46E-01	7.56E-01	7.53E-01	2.62E-03	6.46E-01	8.53E-03	7.55E-01	7.55E-01	4.69E-04	7.55E-01	7.14E-02
**H_2**		3.94E-16	3.46E-15	0.00E+00	1.00E+00	2.56E-02	2.02E-01	3.46E-15	3.46E-15	2.02E-01	2.40E-01	2.39E-01	9.40E-04	2.02E-01	3.39E-03	2.35E-01	2.35E-01	1.98E-04	2.35E-01	2.56E-02
**N_2**		9.33E-01	9.33E-01	1.00E+00	0.00E+00	2.41E-03	6.53E-04	9.33E-01	9.33E-01	6.53E-04	5.69E-04	3.97E-03	9.27E-01	6.53E-04	9.85E-01	5.55E-03	5.55E-03	7.96E-01	5.55E-03	2.41E-03
**H_3PO_4**		3.58E-02	3.58E-02	0.00E+00	0.00E+00	4.04E-06	1.92E-08	3.58E-02	3.58E-02	1.92E-08	1.68E-08	7.07E-06	6.90E-02	1.92E-08	2.04E-03	9.86E-06	9.86E-06	5.75E-02	9.86E-06	4.04E-06
**$(NH_4)_2HPO_4$**		1.90E+03	1.90E+03	3.92E+01	1.04E+01	1.15E+01	4.37E+01	3.79E+01	1.86E+03	4.37E+01	4.37E+01	4.54E+01	1.91E+03	4.37E+01	1.76E+00	3.28E+01	3.34E+01	2.67E-08	3.34E+01	1.15E+01
**Mass Flows**	kg/sec	1.03E+01	1.03E+01	0.00E+00	0.00E+00	1.07E+01	1.17E+01	2.06E-01	1.01E+01	1.17E+01	3.16E-01	3.17E-01	1.26E-01	1.17E+01	4.63E-04	3.17E-01	3.23E-01	7.56E-10	3.23E-01	1.07E+01
**NH_3**	kg/sec	1.27E-14	2.99E-13	0.00E+00	0.00E+00	1.01E-01	5.93E+00	5.99E-15	2.93E-13	5.93E+00	7.96E+00	7.96E+00	1.01E-01	5.93E+00	3.12E-04	5.71E+00	5.83E+00	2.87E-13	5.83E+00	1.01E-01
**H_2**	kg/sec	2.17E-13	1.90E-12	0.00E+00	1.04E+01	5.02E-01	2.57E+01	3.80E-14	1.86E-12	2.57E+01	3.51E+01	3.51E+01	5.02E-01	2.57E+01	1.72E-03	2.47E+01	2.52E+01	1.68E-12	2.52E+01	5.02E-01
**N_2**	kg/sec	1.79E+03	1.79E+03	3.92E+01	0.00E+00	1.65E-01	2.91E-01	3.59E+01	1.76E+03	2.91E-01	2.91E-01	2.04E+00	1.73E+03	2.91E-01	1.75E+00	2.04E+00	2.08E+00	2.37E-08	2.08E+00	1.65E-01
**H_3PO_4**	kg/sec	9.27E+01	9.27E+01	0.00E+00	0.00E+00	3.74E-04	1.16E-05	1.85E+00	9.08E+01	1.16E-05	1.16E-05	4.89E-03	1.74E+02	1.16E-05	4.88E-03	4.89E-03	4.99E-03	2.30E-09	4.99E-03	3.74E-04

**$(NH_4)_2HPO_4$**	kg/sec																			
**Mass Fractions**		5.45E-03	5.45E-03	0.00E+00	0.00E+00	9.33E-01	2.69E-01	5.45E-03	5.45E-03	2.69E-01	7.25E-03	6.98E-03	6.63E-05	2.69E-01	2.63E-04	9.67E-03	9.67E-03	2.83E-02	9.67E-03	9.33E-01
**NH_3**		6.70E-18	1.58E-16	0.00E+00	0.00E+00	8.75E-03	1.36E-01	1.58E-16	1.58E-16	1.36E-01	1.82E-01	1.75E-01	5.28E-05	1.36E-01	1.77E-04	1.74E-01	1.74E-01	1.07E-05	1.74E-01	8.75E-03
**H_2**		1.14E-16	1.00E-15	0.00E+00	1.00E+00	4.37E-02	5.88E-01	1.00E-15	1.00E-15	5.88E-01	8.04E-01	7.73E-01	2.63E-04	5.88E-01	9.80E-04	7.54E-01	7.54E-01	6.30E-05	7.54E-01	4.37E-02
**N_2**		9.46E-01	9.46E-01	1.00E+00	0.00E+00	1.44E-02	6.67E-03	9.46E-01	9.46E-01	6.67E-03	6.67E-03	4.49E-02	9.08E-01	6.67E-03	9.96E-01	6.23E-02	6.23E-02	8.85E-01	6.23E-02	1.44E-02
**H_3PO_4**		4.89E-02	4.89E-02	0.00E+00	0.00E+00	3.25E-05	2.65E-07	4.89E-02	4.89E-02	2.65E-07	2.65E-07	1.08E-04	9.11E-02	2.65E-07	2.78E-03	1.49E-04	1.49E-04	8.62E-02	1.49E-04	3.25E-05
**$(NH_4)_2HPO_4$**		4.10E+00	3.90E+00	6.37E-02	8.94E+00	1.34E+00	1.82E+00	7.81E-02	3.82E+00	1.60E+00	1.62E+00	1.98E+01	3.41E+00	1.56E+00	3.01E-03	1.44E+01	1.47E+01	4.83E-11	1.47E+01	1.34E+00
**Volume Flow**	cum/sec																			

Economic modeling

Capital costs for conventional ammonia production. The cost of capital equipment is estimated using the bare module approach. The capital costs for 10 unit operations are calculated: the 3-bed reactor, 3 coolers, 2 heat exchangers, 2 compressors, a flash vessel for condensation and a cooling tower. To obtain the capital cost, the free on board (FOB) cost is calculated, the L+M factor is used to calculate the bare module cost from the FOB cost and the total investment cost is determined from the bare module cost. The FOB cost is calculated using a power law relationship based on a reference size parameter. A fixed cost is added to the FOB cost to represent the cost of the control systems for each unit operation. For the reactor, a pressure factor of 6.1 is applied in **Eq. S9** below, where the pressure P is in bar:

$$f_p = 0.125 * (P/10) + 0.875 \qquad \text{(S9)}$$

The synthesis catalyst is assumed to cost 15.50 \$/kg.[13] As an example for the reactor bed the FOB cost is calculated using the reactor volume as the basis via **Eq. S10** below. The reactor volume in the conventional case is 121.63 m^3.

$$FOB = (R_{vol}/20\ m^3)^{0.52} * 110{,}000\ USD\ (CEPCI = 1000) \qquad \text{(S10)}$$

Finally, to achieve the bare module (BM) cost, the **Eq. S11** below is used:

$$BM = FOB * p_{factor} * L{+}M + C_{controls} + C_{cat} * 1.1 \qquad \text{(S11)}$$

where the p_{factor} is the pressure factor of 6.1 for 300 bar operation, the L+M factor is 1.495 for stainless steel, the $C_{controls}$ or instrumentation cost is 63,000 USD (CEPCI = 1000) and C_{cat} represents the catalyst cost. The 1.1× term represents any additional field engineering costs required onsite for the equipment. The bare module cost[8] for all the equipment adjusted to 2022 USD through the CEPCI[14] is presented below in **Table S7**.

Table S7. Bare-module capital cost estimates of major equipment in the conventional ammonia production process. See **Figure S8**.

Equipment	Bare Module Cost, 300 bar (USD 2022)	Bare Module Cost, 150 bar (USD 2022)
IC-1 (B1)	111,000	71,700
IC-2 (B5)	80,000	58,400
Three Bed Reactor (including catalyst)	9,700,000	15,722,000
Reactor Preheater (HX1)	297,000	190,000
Product Heat Exchanger (B12)	367,000	232,000
Multistage Feed Compressor	52,425,000	30,877,000
Multistage Recycle Compressor	12,774,000	3,110,000
Product Cooler (PRECOOL)	891,000	681,000
Flash Vessel	53,000	33,550
Cooling Tower	6,000,000	3,900,000

Capital costs for sorption-enhanced ammonia production. Given the preliminary approach to modeling the ammonia-phosphoric acid system in Aspen Plus, it is difficult to accurately assess the costs associated with the absorption system. Expected equipment includes a pre-neutralization stirred tank reactor, an absorption column, a stripping column, the reboiler, pump and condenser associated with the stripping column, flash vessels and pressure changers such as pumps. Equipment in the presence of phosphoric acid at high temperatures and pressures is assumed to be made of Hastelloy given the corrosive nature of phosphoric acid (**Figure S1**). The cost correlations described literature do not always contain an alloy conversion factor that converts carbon steel equipment to Hastelloy equipment. A Hastelloy to carbon steel factor of 2.15 and a Hastelloy to 316 stainless steel factor of 1.47 derived from centrifugal pumps will be used as default conversion factors. Sour water systems are used to estimate the cost of the absorption and stripping columns. The stirred tank reactor and absorber capital costs is assumed to be equivalent to the stripper cost. This assumption is valid given the rapid nature of the neutralization reaction and the additional equipment associated with the stripping tower (reboiler, condenser, pumps, and packing). The

capital costs associated with the relevant equipment in the absorption-enhanced model are presented in **Table S8** below.

Table S8. Bare-module capital cost estimates of major equipment in the sorption-enhanced ammonia production process. See **Figure S9**.

Equipment	Bare Module Cost (USD 2022)
Recirculation Pump	629,000
Makeup Pump	657,000
Recompression Pump[1]	287,000
Absorber and Stirred Tank Reactor	9,050,000
Condenser	9,050,000
Flash Vessel	1,230,000

[1] The recompression pump significantly impacts ammonia production cost in the sorption-enhanced case, but it is only present because the system is depressurized to minimize the amount of phosphoric acid recycled to the synthesis loop. Further validation is needed to understand vapor pressures of phosphoric acid under different states.

Operating costs. Operating costs primarily consist of feedstock costs, utility, labor and maintenance costs. Feedstock costs consist of nitrogen at 0.05 \$/kg, hydrogen at 2 \$/kg and cooling water at 0.00011335 \$/gallon. 100 full time employees are assumed to operate the conventional plant at an average burdened labor cost of 50 \$/man-hr. An additional 10 full-time employees are assumed to be needed for the absorption-based process due to its complexity and unfamiliarity. Utility costs primarily consist of electricity for compression at 50 \$/MWh.

Indirect costs (e.g., engineering, permitting, contingencies) not included in the bare module cost calculation are added to the bare module cost to estimate the total investment cost. Indirect costs are represented as a % of the bare module costs, which are currently evaluated at H2A defaults.[15] The financing structure, tax rates and other generic discounted cash flow parameters are evaluated at H2A defaults.[15]

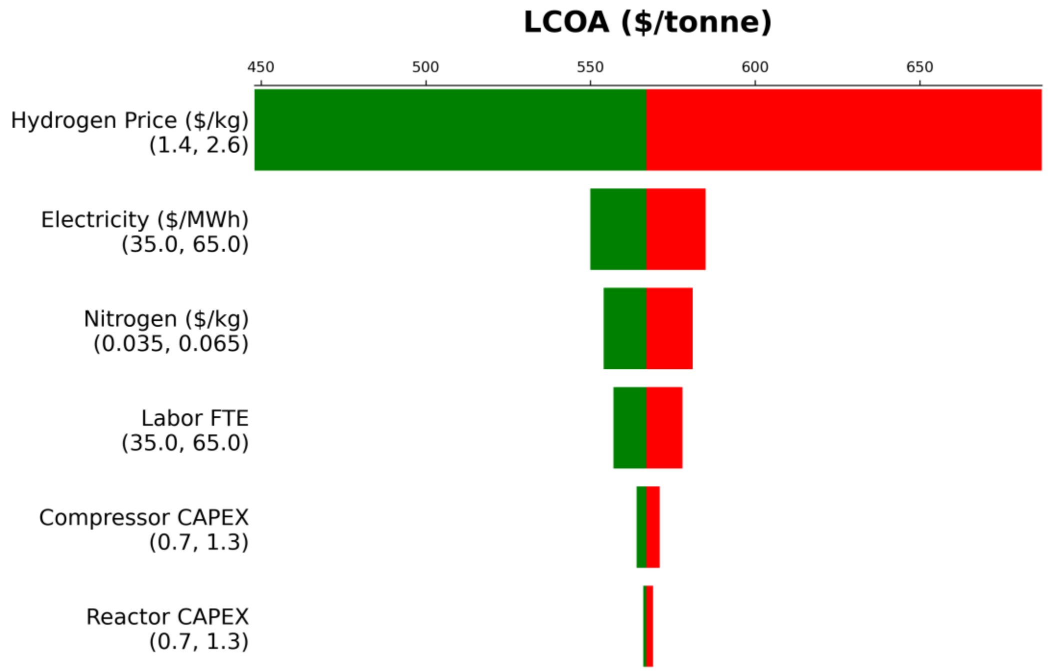

Figure S10. Tornado sensitivity analysis plot of key cost parameters for the conventional 150-bar ammonia synthesis process where each parameter is varied ±30% from the baseline value.

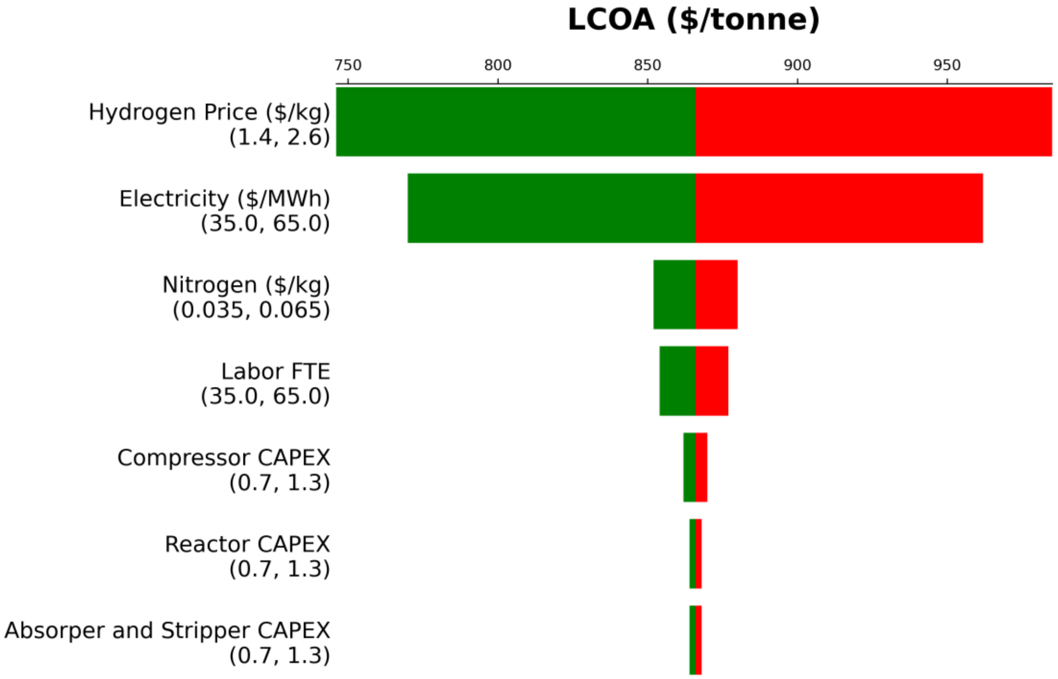

Figure S11. Tornado sensitivity analysis plot of key cost parameters for the sorption-enhanced 150-bar ammonia synthesis process where each parameter is varied ±30% from the baseline value.

References

1. Kresse, G.; Furthmüller, J., Efficient iterative schemes for ab initio total-energy calculations using a plane-wave basis set. *Physical Review B* **1996,** *54* (16), 11169-11186.
2. Kresse, G.; Furthmüller, J., Efficiency of ab-initio total energy calculations for metals and semiconductors using a plane-wave basis set. *Computational Materials Science* **1996,** *6* (1), 15-50.
3. Kresse, G.; Furthmüller, J.; Hafner, J., Theory of the crystal structures of selenium and tellurium: The effect of generalized-gradient corrections to the local-density approximation. *Physical Review B* **1994,** *50* (18), 13181-13185.
4. Kresse, G.; Joubert, D., From ultrasoft pseudopotentials to the projector augmented-wave method. *Physical Review B* **1999,** *59* (3), 1758-1775.
5. Shin, Y. K.; Sengul, M. Y.; Jonayat, A. S. M.; Lee, W.; Gomez, E. D.; Randall, C. A.; Duin, A. C. T. v., Development of a ReaxFF reactive force field for lithium ion conducting solid electrolyte Li1+xAlxTi2−x(PO4)3 (LATP). *Physical Chemistry Chemical Physics* **2018,** *20* (34), 22134-22147.
6. Shan, T.-R.; van Duin, A. C. T.; Thompson, A. P., Development of a ReaxFF Reactive Force Field for Ammonium Nitrate and Application to Shock Compression and Thermal Decomposition. *The Journal of Physical Chemistry A* **2014,** *118* (8), 1469-1478.
7. Thompson, A. P.; Aktulga, H. M.; Berger, R.; Bolintineanu, D. S.; Brown, W. M.; Crozier, P. S.; in 't Veld, P. J.; Kohlmeyer, A.; Moore, S. G.; Nguyen, T. D.; Shan, R.; Stevens, M. J.; Tranchida, J.; Trott, C.; Plimpton, S. J., LAMMPS - a flexible simulation tool for particle-based materials modeling at the atomic, meso, and continuum scales. *Computer Physics Communications* **2022,** *271*, 108171.
8. Woods, D. R., *Rules of Thumb in Engineering Practice*. WILEY-VCH Verlag GmbH & Co. KGaA: Weinheim, Germany, 2007.
9. Morud, J. C.; Skogestad, S., Analysis of instability in an industrial ammonia reactor. *AIChE Journal* **1998,** *44* (4), 888-895.
10. Palys, M. J.; McCormick, A.; Cussler, E. L.; Daoutidis, P., Modeling and Optimal Design of Absorbent Enhanced Ammonia Synthesis. *Processes* **2018,** *6* (7), 91.
11. Nielsen, A.; Kjaer, J.; Hansen, B., Rate equation and mechanism of ammonia synthesis at industrial conditions. *Journal of Catalysis* **1964,** *3* (1), 68-79.
12. Cao, E., Heat Transfer in Process Engineering. 1st Ed. ed.; The McGraw-Hill Companies, Inc.: New York, NY, 2010.
13. Bañares-Alcántara, R.; Dericks III, G.; Fiaschetti, M.; Grünewald, P.; Lopez, J. M.; Tsang, E.; Yang, A.; Ye, L.; Zhao, S. *Analysis of Islanded Ammonia-based Energy Storage Systems*; University of Oxford: Oxford, United Kingdom, 2015.
14. "Chemical Engineering Plant Cost Index". https://www.chemengonline.com/pci.
15. James, B.; Colella, W.; Moton, J.; Sauer, G.; Ramsden, T. *PEM Electrolysis H2A Production Case Study Documentation*; National Renewable Energy Laboratory (NREL), Golden, CO: 2013.
